# Supplementary material for: MicroRNA governs bistable cell differentiation and lineage segregation via a noncanonical feedback
Source: Mol Syst Biol. 2021 Apr 23;17(4):e9945. doi: 10.15252/msb.20209945 (PMC8062999; doi:10.15252/msb.20209945)
Supplement: Supplementary file 1 — Appendix [file MSB-17-e9945-s005.pdf]

Appendix

MicroRNA governs bistable cell differentiation and lineage segregation via a noncanonical feedback

Chung-Jung Li[1,2], Ee Shan Liau[1,2], Yi-Han Lee[2], Yang-Zhe Huang[2], Ziyi Liu[3], Andrew Willems[3], Victoria Garside[4], Edwina McGlinn[4], Jun-An Chen[1,2,5*], Tian Hong[6,7,*]

1. Molecular and Cell Biology, Taiwan International Graduate Program, Academia Sinica and Graduate Institute of Life Science, National Defense Medical Center, Taipei, Taiwan.

2. Institute of Molecular Biology, Academia Sinica, Taipei, Taiwan.

3. Genome Science and Technology Program, The University of Tennessee, Knoxville, Tennessee, USA.

4. EMBL Australia, Australian Regenerative Medicine Institute, Monash University, Clayton, Victoria, Australia.

5. Neuroscience Program Academia Sinica, Taipei, Taiwan.

6. Department of Biochemistry & Cellular and Molecular Biology, The University of Tennessee, Knoxville, Tennessee, USA.

7. National Institute for Mathematical and Biological Synthesis, Knoxville, Tennessee, USA.

Table of Contents

1. Mathematical models and analysis 3

1.1 List of all models 3

Appendix Table S1 3

1.2 Transcriptional cross repression (T-CR) Model 3

1.3 Transcriptional unilateral repression (T-UR) Model 5

1.4 Transcriptional unilateral repression with miRNA regulation (Tmi-UR) Model 6

1.5 Transcriptional unilateral repression with transcription and miRNA mediated feedback (Tmi-FB) Model 8

1.6 Performance evaluation of spatiotemporal models. 8

1.7 mRNA-miRNA model with one binding site (mmi-1 Model) 9

1.8 mRNA-miRNA model with two binding sites (mmi-2 Model) 11
1.8.1 Model construction and simplification 11
1.8.2 Analysis of the number of steady states 14
1.8.3 Numerical experiments for bistable switches 16
Appendix Table S2 17
Appendix Figure S1 18
Appendix Figure S2 19
1.9 mRNA-miRNA model with three binding sites (mmi-3 Model) 20
Appendix Figure S3 20
1.10 Performance of bistability predictions with reduced association constant 21
1.11 Estimate of realistic biological circuits described by mmi-2 and mmi-3 Models 21
Appendix Figure S4 22
Appendix Table S3 23
1.12 Effects of a competitor mRNA on bistable switches governed by mmi-2 Model 23
Appendix Figure S5 24
1.13 mRNA-miRNA with noncanonical feedback and morphogen gradients (mmi-S Model) 25
Appendix Figure S6 25
1.14 List of parameter values and ranges for random sampling 26
Appendix Table S4 27
Appendix Table S5 28
2. Additional Information of Resources 29
2.1 List of key reagents and resources 29
Appendix Table S6 29
2.2 Primers for 3´ UTR mutants 31
Appendix Table S7 31
2.3 Primers for genotyping 31
Appendix Table S8 31
2.4 Sequence for miRNA sponge 32
Appendix Figure S7 33
Appendix References 35

1. Mathematical models and analysis

1.1 List of all models

Table S1 summarizes the information of all mathematical models in this study. The subsequent sections describe these models in detail.

Appendix Table S1. List of all models

Model abbreviation	Model full name	Morphogen gradient	Presenting figure	Text with details
T-CR	Transcriptional cross repression	Included	Figure EV2	Supplementary Text 1.2
T-UR	Transcriptional unilateral repression	Included	Figure EV2	Supplementary Text 1.3
Tmi-UR	Transcriptional unilateral repression with miRNA regulation	Included	Figure EV3	Supplementary Text 1.4
Tmi-FB	Transcriptional unilateral repression with transcription and miRNA mediated feedback	Included	Figure EV3	Supplementary Text 1.5
mmi-1	mRNA-miRNA with one binding site	Not included	Figure 4	Supplementary Text 1.7
mmi-2	mRNA-miRNA with two binding sites	Not included	Figure 4	Supplementary Text 1.8
mmi-3	mRNA-miRNA with three binding sites	Not included	Appendix Figure S5	Supplementary Text 1.9
mmi-S	mRNA-miRNA with noncanonical feedback and morphogen gradients	Included	Figure 7	Supplementary Text 1.11

1.2 Transcriptional cross repression (T-CR) Model

To understand the segregation of $Hoxa5^{on}Hoxc8^{off}$ and $Hoxa5^{off}Hoxc8^{on}$ motor neurons (MNs), we first considered a canonical model of transcriptional cross repression (T-CR) between Hoxa5 and Hoxc8. The model consists of 40 compartments (cells) describing discretized space spanning the rostral-caudal axis of the developing spinal cord. In each compartment, the lineage decision of each cells is governed by the following ordinary differential equations (ODEs)

$$\frac{dR_5}{dt} = s_5^0 + s_5 \frac{(A(t)/K_{5A})^{n_{5A}}}{1 + (A/K_{5A})^{n_{5A}} + (P_8/K_{58})^{n_{58}}} - k_5 R_5 \tag{1.2.1a}$$

$$\frac{dR_8}{dt} = s_8^0 + s_8 \frac{(F(t)/K_{8F})^{n_{8F}}}{1 + (F/K_{8F})^{n_{8F}} + (P_5/K_{85})^{n_{85}}} - k_8 R_8 \tag{1.2.1b}$$

$$\frac{dP_5}{dt} = l_5^0 R_5 - P_5 \tag{1.2.1c}$$

$$\frac{dP_8}{dt} = l_8^0 R_8 - P_8. \tag{1.2.1d}$$

Here, R_5, P_5, R_8, P_8 represent the concentration of *Hoxa5* mRNA, Hoxa5 protein, *Hoxc8* mRNA and Hoxc8 protein, respectively. s_5^0 and s_8^0 are the transcription factors independent (basal) production rate constants of *Hoxa5* mRNA and *Hoxc8* mRNA, respectively. K_{XY} represents the apparent threshold of activation or inhibition of mRNA X by transcription factor Y, and n_{XY} describes the nonlinearity of the same transcriptional regulation. k_5 and k_8 are the degradation rate constants of *Hoxa5* mRNA and *Hoxc8* mRNA, respectively. l_5^0 and l_8^0 are the translation rate constants of free forms of *Hoxa5* mRNA and *Hoxc8* mRNA, respectively. Although it is unnecessary to model protein and mRNA dynamics separately for the demonstration of lineage decision and boundary formation in this case, we considered mRNA and protein dynamics explicitly to keep the model consistent with other models in this study.

To describe the distribution of RA and FGF across the tissue domain, we first considered a one-dimensional tissue domain with a length of L, and a reaction-diffusion equation describing a morphogen concentration M that is a function of time and space

$$\frac{dM}{dt} = D \frac{d^2 M}{dx^2} - kM. \tag{1.2.2}$$

Here, D is the diffusion coefficient (length squared per unit time). k is the degradation rate constant (the reciprocal of unit time). As a biological constrain, we assumed that there is a source of morphogen production at the boundary $x = 0$, and that the boundary $x = L$ has a relatively low concentration of the morphogen.

At steady state, the distribution M_s is described by

$$0 = D \frac{d^2 M_s}{dx^2} - kM_{s\,.} \tag{1.2.3}$$

Suppose $u(x)$ is the solution to this second-order ODE. We have the general solution

$$u(x) = Ae^{x} + Be^{-x}, \tag{1.2.4}$$

where A and B are constants that depend on the boundary conditions. Since the solution should hold for tissues that are very long relative to the size of a cell ($L \to \infty$), we must have $A = 0$. If $A \neq 0$, then the e^{x} term will diverge to infinity as $x \to \infty$ while e^{-x} approaches zero, and the solution will be inconsistent with the biological constraint.

For boundary conditions, we assumed constant flux at one end of the tissue and a constant low concentration at the other end:

$$\frac{dM(0,t)}{dx} = \sigma, \quad t \geq 0 \tag{1.2.5a}$$

$$M(L,t) = \varepsilon, \quad t \geq 0. \tag{1.2.5b}$$

Given these boundary conditions, it follows from Eq 1.2.4 that

$$B = \sigma = \varepsilon e^{L}. \quad (1.2.6)$$

Therefore, the steady state distribution of the morphogen is

$$M_s = u(x) = \sigma e^{-x}. \quad (1.2.7)$$

We conclude that it is reasonable to use an exponential function to describe a morphogen gradient across a one-dimensional tissue domain.

With the assumption above, we considered quasi-steady-state concentrations of RA and FGF (described by A and F respectively), and their dynamics are governed by $\partial_t M = D\partial_x^2 M - kM$, where M is the concentration of RA or FGF (A or F). We assumed that the RA and FGF are synthesized at the rostral boundary and caudal boundary, respectively. We assumed that D and k are relatively fast with respect to intracellular kinetics. Based on the earlier analysis and assumptions, we approximated A or F by steady solution of the reaction-diffusion system which has an antiparallel, exponential pattern of RA and FGF along the RC axis. We further approximated the RC axis of the tissue by 40 compartments, and dynamics of A or F in the ith compartment along the axis are approximated by

$$\frac{dA_i}{dt} = \gamma_A\left(m_A e^{\sigma_A i} - A_i\right) \quad (1.2.8a)$$

$$\frac{dF_i}{dt} = \gamma_F\left(m_F e^{\sigma_F (L-i)} - F_i\right). \quad (1.2.8b)$$

γ_A and γ_F represent the timescales of the RA and FGF dynamics (assumed to be 1). σ_A and σ_F are length scale constants (assumed to be 0.01) determined by D and k of the morphogens. L is the width of the modeled space (40 units). m_A and m_F are the levels of RA at the rostral boundary and FGF at the caudal boundary of the modeled space, respectively. These two parameters were assumed to be time dependent to reflect realistic RA and FGF dynamics that are most plausible during development: m_A and m_F were assumed to increase abruptly during the early spinal cord development (the increase of m_A from 0 to 2.1 was followed by the increase of m_F from 0 to 2.3), and then gradually declined (m_A and m_F were reduced to 65% of their maximal values, and γ_A and γ_F were decreased from 1 to 0.005). The assumed dynamics can be explained by the rapid activation of RA synthesis at the rostral end of the embryo, followed by FGF synthesis activation at the caudal end, and the subsequent expansion of the embryo along the rostral-caudal axis. Although the dynamics of RA and FGF are difficult to measure in the mouse spinal cord, transient morphogen signals are consistent with previous observations (Ensini *et al*, 1998; Mazzoni *et al*, 2013). In addition, we considered temporal fluctuations of RA and FGF signaling by introducing a moderate amount of noise when we simulated Eq 1.2.8. The positional information encoded by steady state RA concentration was previously reported to be noisy and shallow in zebrafish (Sosnik *et al*, 2016). Each derivative in Eq 1.2.8 has an additional noisy input εX, where X is the concentration of RA or FGF (A or F), and $\varepsilon = 0.001 \cdot N(0,1)$. $N(0,1)$ is a random number drawn from a normal distribution with mean of 0 and unit variance. During the simulations, a random number was drawn every 5 time units after time 300.

1.3 Transcriptional unilateral repression (T-UR) Model

Experimental evidence has suggested that Hoxc8 inhibits Hoxa5 in a unilateral fashion (Dasen *et al*, 2005; Philippidou & Dasen, 2013) (this study). Therefore, the T-CR Model is unlikely valid. To model the MN

differentiation with a more realistic gene regulatory network (GRN), we modified Eq 1.2.1 by simplify removing the repression of *Hoxc8* transcription by Hoxa8, and considered the following ODEs to describe the Hoxa5 and Hoxc8 dynamics

$$\frac{dR_5}{dt} = s_5^0 + s_5 \frac{(A(t)/K_{5A})^{n_{5A}}}{1 + (A/K_{5A})^{n_{5A}} + (P_8/K_{58})^{n_{58}}} - k_5 R_5 \tag{1.3.1a}$$

$$\frac{dR_8}{dt} = s_8^0 + s_8 \frac{(F(t)/K_{8F})^{n_{8F}}}{1 + (F/K_{8F})^{n_{8F}}} - k_8 R_8 \tag{1.3.1b}$$

$$\frac{dP_5}{dt} = l_5^0 R_5 - P_5 \tag{1.3.1c}$$

$$\frac{dP_8}{dt} = l_8^0 R_8 - P_8. \tag{1.3.1d}$$

The descriptions of variables and parameters are identical to the T-CR Model. Several adjustments of parameter values were made to achieve clear segregation of Hoxa5^{on} and Hoxc8^{on} MNs in the presence of noise-free morphogen signals.

1.4 Transcriptional unilateral repression with miRNA regulation (Tmi-UR) Model

To consider post-transcriptional regulation, we incorporated two miRNAs, miR-27 and miR-196, into the T-UR model. miR-27 and miR-196 control the expression of Hoxa5 and Hoxc8 respectively (Li *et al*, 2017; Wong *et al*, 2015). The modeling framework is similar to previous studies concerning miRNA regulations (Lu *et al*, 2013; Riba *et al*, 2014). In this framework, miRNAs inhibit protein production via inducing mRNA degradation and translational repression upon binding to the 3' UTR of the target mRNAs. The 3' UTR of *Hoxa5* mRNA has three conserved putative binding sites for miR-27, whereas that of the *Hoxc8* mRNA has four conserved putative binding sites for miR-196 (this study). The intracellular system is described by the following ODEs

$$\frac{dR_5}{dt} = s_5^0 + s_5 \frac{(A(t)/K_{5A})^{n_{5A}}}{1 + (A/K_{5A})^{n_{5A}} + (P_8/K_{58})^{n_{58}}} - k_5 (R_5 - \sum_{i=1}^{3} \binom{3}{i} C_5^i) - \sum_{i=1}^{3} k_5^i \binom{3}{i} C_5^i \tag{1.4.1a}$$

$$\frac{dR_8}{dt} = s_8^0 + s_8 \frac{(F(t)/K_{8F})^{n_{8F}}}{1 + (F/K_{8F})^{n_{8F}}} - k_8 (R_8 - \sum_{i=1}^{4} \binom{4}{i} C_8^i) - \sum_{i=1}^{4} k_8^i \binom{4}{i} C_8^i \tag{1.4.1b}$$

$$\frac{dP_5}{dt} = l_5^0 (R_5 - \sum_{i=1}^{3} \binom{3}{i} C_5^i) + \sum_{i=1}^{3} l_5^i \binom{3}{i} C_5^i - P_5 \tag{1.4.1c}$$

$$\frac{dP_8}{dt} = l_8^0 (R_8 - \sum_{i=1}^{4} \binom{4}{i} C_8^i) + \sum_{i=1}^{4} l_8^i \binom{4}{i} C_8^i - P_8 \tag{1.4.1d}$$

$$\frac{dr_2}{dt} = s_2 \frac{1}{1 + (A/K_{2A})^{n_{2A}}} - k_2(r_2 - \sum_{i=1}^{3} i \binom{3}{i} C_5^i) - \sum_{i=1}^{3} i k_2^i \binom{3}{i} C_5^i \tag{1.4.1e}$$

$$\frac{dr_9}{dt} = s_9 - k_9(r_9 - \sum_{i=1}^{4} i \binom{4}{i} C_8^i) - \sum_{i=1}^{4} i k_9^i \binom{4}{i} C_8^i. \tag{1.4.1f}$$

Here, R_5 and R_8 are the total concentrations of *Hoxa5* and *Hoxc8* mRNAs, respectively, including those bound by miRNAs and free mRNAs. r_2 and r_9 are the total concentrations of miR-27 and miR-196, respectively. l_5^i and k_5^i are the translation rate constant and degradation rate constant of *Hoxa5* mRNA when i numbers of miR-27 bind to it, respectively. k_2^i is the degradation rate constant of miR-27 in these complexes. l_8^i and k_8^i are the translation rate constant and degradation rate constant of *Hoxc8* mRNA when i numbers of miR-196 bind to it, respectively. k_9^i is the degradation rate constant of miR-196 in these complexes. s_2 and s_9 are the maximal production rate constants of miR-27 and miR-196 respectively. k_2 and k_9 are the degradation rate constants of free forms of miR-27 and miR-196 respectively.

$\sum_{i=1}^{3} i \binom{3}{i} C_5^i = 3C_5^1 + 2 \cdot 3C_5^2 + 3C_5^3$, and this represents the total amount of miR-27 bound to *Hoxa5* mRNA. Each term of this summation describes $\binom{3}{i}$ scenarios in which i number of miRNA molecules bind to 3 possible binding sites that each *Hoxa5* mRNA has. $\sum_{i=1}^{3} \binom{3}{i} R_{5i} = 3C_5^1 + 3C_5^2 + C_5^3$, and this represents the total amount of complex formed by miR-27 and *Hoxa5* mRNA. The complexes for miR-196 bound *Hoxc8* mRNA are defined similarly: $\sum_{i=1}^{4} \binom{4}{i} C_8^i = 4C_8^1 + 6C_8^2 + 4C_8^3 + C_8^4$, and so are the total amount of miR-196 bound to *Hoxc8* mRNA: $\sum_{i=1}^{4} i \binom{4}{i} C_8^i = 4C_8^1 + 2 \cdot 6C_8^2 + 3 \cdot 4C_8^3 + 4C_8^4$. The concentrations of these complexes are determined by the following algebraic equations

$$C_5^1 = \kappa_5^1 (r_2 - \sum_{i=1}^{3} i \binom{3}{i} C_5^i)\,(R_5 - \sum_{i=1}^{3} \binom{3}{i} C_5^i) \tag{1.4.2a}$$

$$C_5^i = \kappa_5^i (r_2 - \sum_{j=1}^{3} j \binom{3}{j} C_5^j)\, C_5^{i-1} \qquad i = 2,3 \tag{1.4.2b}$$

$$C_8^1 = \kappa_8^1 (r_9 - \sum_{i=1}^{4} i \binom{4}{i} C_8^i)\,(R_8 - \sum_{i=1}^{4} \binom{4}{i} C_8^i) \tag{1.4.2d}$$

$$C_8^i = \kappa_8^i (r_9 - \sum_{j=1}^{4} j \binom{4}{j} C_8^j)\, C_8^{i-1} \qquad i = 2,3,4. \tag{1.4.2e}$$

κ_5^i and κ_8^i are the inverse of the dissociation constants (i.e. association constants) for complex formation of C_5^i and C_8^i respectively.

Similar modeling strategies have been used to model miRNA mediated feedforward loops, as well as feedback loops involving transcriptional inhibition of miRNAs by transcription factors (Lu *et al.*, 2013; Riba *et al.*, 2014). In this study, we took advantage of this established framework for building all of our

models involving miRNAs. However, we addressed two key limitations of the previous modeling framework. First, instead of obtaining concentrations of the complexes with an approximation (Lu *et al.*, 2013), we calculated them explicitly for better accuracy (e.g. Eq 1.4.2). Secondly, the degradation rate constants of each mRNA or miRNA in multiple complexes (i.e. k_X^i where X is any mRNA or miRNA, and $i \geq 1$) were assumed to be identical in a previous study (Riba *et al.*, 2014). This assumption is inconsistent with several experimental studies (de la Mata *et al*, 2015; Ghini *et al*, 2018; Grimson *et al*, 2007). A previous modeling study by Tian et al. relaxed this assumption in certain parameter region, and reported a possible bistable arising from purely post-transcriptional reactions (Tian *et al*, 2016). However, systematic analysis of k_X^i and comparison with experimental data were not performed. In this study, we relaxed this assumption with systematic analysis of the effect of differential k_X^i in multiple mRNA-miRNA complexes (1.7-1.9).

1.5 Transcriptional unilateral repression with transcription and miRNA mediated feedback (Tmi-FB) Model

The Tmi-FB model extends the Tmi-UR model by assuming transcriptional repression of miR-27 by Hoxa5, and repression of miR-196 by Hoxc8, i.e. replace Eq 1.4.1e and 1.4.1f by the following ODEs

$$\frac{dr_2}{dt} = s_2 \frac{1}{1 + (A/K_{2A})^{n_{2A}} + (P_5/K_{25})^{n_{25}}} - k_2(r_2 - \sum_{i=1}^{3} i \binom{3}{i} C_5^i) - \sum_{i=1}^{3} ik_5^i \binom{3}{i} C_5^i \qquad (1.5.1a)$$

$$\frac{dr_9}{dt} = s_9 \frac{1}{1 + (P_8/K_{98})^{n_{98}}} - k_9(r_9 - \sum_{i=1}^{4} i \binom{4}{i} C_8^i) - \sum_{i=1}^{4} ik_8^i \binom{4}{i} C_8^i. \qquad (1.5.1b)$$

All other equations are identical to Eq 1.2.8, Eq 1.4.1 and Eq 1.4.2. A hypothetical inhibition of miR-27 by Hoxa5 is described by K_{25} (threshold) and n_{25} (cooperativity). Similarly, a hypothetical inhibition of miR-196 by Hoxc8 is described by K_{98} (threshold) and n_{98} (cooperativity). Neither of these transcriptional inhibitions is supported by experimental data.

1.6 Performance evaluation of spatiotemporal models.

To evaluate T-CR, T-UR, Tmi-UR and Tmi-FB Models in terms of their capacity of governing cell lineage segregation at the tissue boundary and their consistency with experimental observations, we performed numerical simulations and analysis of the four models with the following scheme:

1. **Simulation procedure**: We used a 10×40 grid representing 400 cells for each model. These cells receive various levels of RA and FGF signals based on their indices of columns which represent the positions along the RC axis. In each simulation, RA and FGF signals in all cells were rapidly increased at time 50 and 100 respectively to its maximum levels (see 1.2), and then starting from time 150 (RA) and time 200 (FGF) slowly decreased to 65% of their maximum levels. This dynamic profile reflects the possible morphogen dynamics during development, which has not been measured experimentally. The RA and FGF signals were also subject to moderate temporal fluctuations in all cells (see 1.2). At time 1000, all state variables have approached their steady states. Time evolutions of the lineage defining molecules Hoxa5 and Hoxc8 proteins obtained from four representative simulations are shown in Movies EV1-EV4.

2. **Parameter sampling**: To ensure that the performance evaluations were not sensitive to the choice of parameters, we chose 10000 parameter sets randomly for each model, except for a few parameters which were assumed to be the same constants across all models. For gene regulations common to multiple models, the corresponding parameter values were chosen from the same log-normal distributions with μ and σ values listed in Table S4. Because we were interested in the upper bound of performance of each model, all nonlinearity parameter (n) was set to 6, which is biological plausible under physiological conditions. For each model, one representative parameter set was chosen and adjusted manually to show simulation trajectories and patterns of cellular distributions. Other analyses were performed based on the results from all sampled parameter sets.

3. **Performance of lineage segregation**: We analyzed the distributions of all simulated molecules at time 1000. To quantify the lineage decision performance at the tissue boundary, we used the Transition Width (W) defined as the number of columns in the 10×40 grid where one or more elements (cells) have undetermined lineage at the final time point (1000). To define the lineage decision, we first define molecule X is expressed in a cell when its level at time 1000 is higher than 5% of the maximum level of X across all cells in the domain at the same time point. If a column of cells in the domain satisfies at least one of the following conditions, the column is counted toward W: 1) at least one cell expresses both Hoxa5 and Hoxc8; 2) at least one cell expresses neither Hoxa5 nor Hoxc8; 3) the column has both Hoxa5^{on}Hoxc8^{off} and Hoxa5^{off}Hoxc8^{on} cells. Because this procedure does not guarantee a meaningful boundary, we further constrain W by enforcing that at least one adjacent column pair has a difference in either Hoxa5 and Hoxc8 higher than 35% of the overall change across the domain (i.e. between rostral end and caudal end). If this condition is not satisfied, W is set to the maximum (40). A model has a desirable performance when $W = 0$. Out of the four models, T-CR and Tmi-FB Models had some of the sampled parameter sets that produced transition width of zero (4.4% and 1.97% respectively out of 10000), whereas the other two models did not have any parameter set that achieved this performance.

3. **Difference between segregations of mRNAs and proteins**. Because we observed a clear cell-to-cell segregation of Hoxa5 and Hoxc8 proteins at the boundary, but not their mRNAs, a key metric to evaluate the models is the differential segregation degrees of mRNAs and proteins. To describe this performance, we used a segregation index $S = \frac{\sup(\Delta R_5)+\sup(\Delta R_5)}{\sup(\Delta P_5)+\sup(\Delta P_5)}$, where ΔX is a vector representing the ratios of mean levels of X between all 39 adjacent column pairs in the domain. We define that a model has a desirable performance when $S < 0.2$. Out of the four models, Tmi-UR and Tmi-FB Models had some of the sampled parameter sets that produced $S < 0.2$ (0.018% and 11.26% respectively out of 10000 sets), whereas the other two models did not have any parameter set that achieved this performance.

Using the criterion based on W and S, 190 parameter sets were selected, and all of them were from the Tmi-FB Model. By analyzing the miR-196 distributions from the simulations with these parameter sets, we found that all of the simulations had very low levels (<0.01) of miR-196 at the caudal boundary of this domain, an observation inconsistent with published experimental data (Kloosterman *et al*, 2006; Wong *et al.*, 2015).

1.7 mRNA-miRNA model with one binding site (mmi-1 Model)

To analyze mRNA-miRNA interactions at a more fundamental level, we revisit the biochemical reactions underpinning models describing the interactions, e.g. the Tmi-UR Model. We first considered a model describing concentrations of a miRNA and an mRNA (target) with one binding site for the miRNA. The full reaction network is as follows:

$$\xrightarrow{s_R} R \qquad R \xrightarrow{k_R^0} \emptyset \qquad \xrightarrow{s_r} r \qquad r \xrightarrow{k_r^0} \emptyset \qquad R + r \xleftrightarrow{K = k_C^{\text{off}}/k_C^{\text{on}}} C \qquad C \xrightarrow{k_r^1} R \qquad C \xrightarrow{k_R^1} r.$$

Here, R is the concentration of the free mRNA. r is the concentration of the free miRNA. C is the concentration of the partially double-stranded RNA molecule formed by partial complementarity of one miRNA molecule and one mRNA molecule. For simplicity, this type of RNA molecule is also called complex in this study. s_R is the synthesis rate constant of mRNA. k_R^0 is the degradation rate constant of free mRNA. s_r is the synthesis rate constant of miRNA. k_r^0 is the degradation rate constant of free miRNA. k_R^1 is the degradation rate constant of mRNA in the complex. k_r^1 is the degradation rate constant of miRNA in the complex. K is the dissociation constant of the binding of miRNA to mRNA. It is the ratio between the rate constant for complex dissociation (k_C^{off}) and that for miRNA-mRNA binding (k_C^{on}).

We applied Chemical Reaction Network Theory (CRNT) with these reactions and found that the system cannot be bistable (Feinberg, 2019). To confirm this result and to provide a foundation for more complex models, we used the following ODEs to describe the reactions with the law of mass action

$$\frac{dR}{dt} = s_R - k_R^0 R - k_C^{\text{on}} R r + k_C^{\text{off}} C + k_r^1 C \qquad (1.7.1a)$$

$$\frac{dr}{dt} = s_r - k_r^0 R - k_C^{\text{on}} R r + k_C^{\text{off}} C + k_R^1 C \qquad (1.7.1b)$$

$$\frac{dC}{dt} = k_C^{\text{on}} R r - k_C^{\text{off}} C - k_r^1 C - k_R^1 C. \qquad (1.7.1c)$$

We next used total quasi-steady state approximation (tQSSA) to reduce the number of ODEs (Borghans *et al*, 1996; Ciliberto *et al*, 2007). This approximation assumes that the binding and unbinding rate constants $k_C^{\text{off}}, k_C^{\text{on}}$ are much greater than other rate constants. As such, the reaction $R + r \leftrightarrow C$ is always at steady state any at given t. The new ODEs describe slow reactions concerning the total concentrations of mRNA and miRNA only

$$\frac{dR_{\text{T}}}{dt} = s_R - k_R^0 R - k_R^1 C \qquad (1.7.2a)$$

$$\frac{dr_{\text{T}}}{dt} = s_r - k_r^0 r - k_r^1 C. \qquad (1.7.2b)$$

Here, R_{T} and r_{T} represent the concentrations of the mRNA and the miRNA, respectively. The concentrations of individual molecules r, R and C are determined by the following equation

$$k_C^{\text{off}} C = k_C^{\text{on}} R r$$

$$\Leftrightarrow \quad KC = Rr. \qquad (1.7.3)$$

Eq 1.7.3 indicates that $\{R, r, C\}$ are at steady state at any given t. This implies that these three variables are bounded for any bounded $\{R_\mathrm{T}, r_\mathrm{T}\}$ at any given t. We next show that this system described by Eq 1.7.2 and Eq 1.7.3 cannot be bistable. A bistable system is defined as a system with two stable steady states and one unstable steady state. The steady state of system is governed by

$$0 = s_R - k_R^0 R - k_R^1 C \tag{1.7.4a}$$

$$0 = s_r - k_r^0 r - k_r^1 C \tag{1.7.4b}$$

$$0 = KC - Rr. \tag{1.7.4c}$$

Solving Eq 1.7.4a and b for R and r respectively, and substituting them in Eq 1.7.4c yields

$$\frac{k_r^1 k_R^1 C^2 - (K k_R^0 k_r^0 + k_r^1 s_R + k_R^1 s_r)C + s_R s_r}{k_R^0 k_r^0} = 0. \tag{1.7.5}$$

Since the numerator is a quadratic polynomial and the denominator is a positive constant, the equation has at most two real solutions. Therefore, the system described by Eq 1.7.2 and Eq 1.7.3 cannot be bistable. We concluded that the elementary interactions involving a miRNA and an mRNA with only one miRNA binding site do not allow bistable switches.

1.8 mRNA-miRNA model with two binding sites (mmi-2 Model)

1.8.1 Model construction and simplification

We next considered a model describing concentrations of a miRNA and an mRNA (target) with two binding sites for the miRNA. The full reaction network is as follows:

$$\xrightarrow{s_R} R \qquad R \xrightarrow{k_R^0} \emptyset \qquad \xrightarrow{s_r} r \qquad r \xrightarrow{k_r^0} \emptyset \qquad R + r \xleftrightarrow{K_1 = k_{C_1}^{\mathrm{off}}/k_{C_1}^{\mathrm{on}}} C_1 \qquad C_1 + r \xleftrightarrow{K_2 = k_{C_2}^{\mathrm{off}}/k_{C_2}^{\mathrm{on}}} C_2$$

$$C_2 \xrightarrow{2k_r^2} C_1 \qquad C_2 \xrightarrow{k_R^2} 2r \qquad C_1 \xrightarrow{k_R^1} r \qquad C_1 \xrightarrow{k_r^1} R.$$

Here, C_1 is the concentration of the partially double-stranded RNA molecule formed by partial complementarity of one miRNA and one mRNA molecules (1:1 complex). Note that this complex has two forms because of the two binding sites. For simplicity, we assumed that the two binding sites are equivalent throughout this study. C_2 is the concentration of the partially double-stranded RNA molecule formed by partial complementarity of one miRNA and one mRNA molecules (2:1 complex). K_1 and K_2 are the dissociation constants for the two complexes respectively. k_R^1 is the degradation rate constant of mRNA in the 1:1 complex. k_r^1 is the degradation rate constant of miRNA in the 1:1 complex. k_R^2 is the degradation rate constant of mRNA in the 2:1 complex. k_r^2 is the degradation rate constant of miRNA in the 2:1 complex.

Other notations are described in 1.7. Similar to the mmi-1 Model, a key underlying assumption is that miRNA and mRNA are degraded independently from their partially double-stranded forms.

We describe the reactions with the law of mass action and the tQSSA using the following differential algebraic equations

$$\frac{dR_{\mathrm{T}}}{dt} = s_R - k_R^0 R - 2k_R^1 C_1 - k_R^2 C_2 \quad (1.8.1a)$$

$$\frac{dr_{\mathrm{T}}}{dt} = s_r - k_r^0 r - 2k_r^1 C_1 - 2k_r^2 C_2 \quad (1.8.1b)$$

$$0 = Rr - K_1 C_1 \quad (1.8.1c)$$

$$0 = C_1 r - K_2 C_2 \quad (1.8.1d)$$

$$0 = R + 2C_1 + C_2 - R_{\mathrm{T}} \quad (1.8.1e)$$

$$0 = r + 2C_1 + 2C_2 - r_{\mathrm{T}}. \quad (1.8.1f)$$

Here, Eq 1.8.1a and b describe slow processes (synthesis and degradation) that control the changes of the state variables, whereas Eq 1.8.1c-f govern the concentrations of molecules that are determined by fast processes, i.e. binding and unbinding. Eq 1.8.1c-f indicate that $\{R, r, C_1, C_2\}$ are at steady state at any given t. This implies that these four variables are bounded for any bounded $\{R_{\mathrm{T}}, r_{\mathrm{T}}\}$ at any given t. To reduce the number of parameters, we considered a scaled independent variable $\tau = k_R^0 t$. We assumed that $k_R^0, s_R > 0$ (non-zero production rate and degradation rate constants of R), and we defined $\gamma = k_r^0/k_R^0$, $\mu = s_r/s_R$, $k_R^1 = a_1 k_R^0$, $k_R^2 = a_2 k_R^0$, $\bar{s}_{\mathrm{R}} = s_R/k_R^0$, $\bar{R}_{\mathrm{T}} = R_{\mathrm{T}}/\bar{s}_R$, $\bar{r}_{\mathrm{T}} = r_{\mathrm{T}}/\bar{s}_R$, $\bar{C}_1 = C_1/\bar{s}_R$, $\bar{C}_2 = C_2/\bar{s}_R$. The ODEs in Eq 1.8.1 can be rewritten as follows

$$k_R^0 \bar{s}_R \frac{d\bar{R}_{\mathrm{T}}}{d\tau} = k_R^0 \bar{s}_R - k_R^0 \bar{s}_R (\bar{R} + 2a_1 \bar{C}_1 + a_2 \bar{C}_2)$$

$$k_R^0 \bar{s}_R \frac{d\bar{r}_{\mathrm{T}}}{d\tau} = s_r - k_r^0 \bar{s}_R (\bar{r} + 2b_1 \bar{C}_1 + 2b_2 \bar{C}_2)$$

$$\Leftrightarrow \quad \frac{d\bar{R}_{\mathrm{T}}}{d\tau} = 1 - (\bar{R} + 2a_1 \bar{C}_1 + a_2 \bar{C}_2) \quad (1.8.2a)$$

$$\frac{d\bar{r}_{\mathrm{T}}}{d\tau} = \mu - \gamma(\bar{r} + 2b_1 \bar{C}_1 + 2b_2 \bar{C}_2). \quad (1.8.2b)$$

Here, a_1 and a_2 are the fold-changes of degradation rate constant of mRNA upon miRNA binding with respect to free mRNA. b_1 and b_2 are the fold-changes of degradation rate constant of miRNA upon mRNA binding with respect to free miRNA.

We assumed that the miRNA regulates the mRNA when the mRNA is actively synthesized (an 'on-state' of the gene). Suppose at a steady state where no miRNA is produced ($s_r = r_{\mathrm{T}} = C_1 = C_2 = 0$), and R_{T} represents a molar concentration in the range of $(10^{-9}, 10^{-7})$ M at this steady state (Lahtvee *et al*, 2017),

then the approximate range of $\bar{s}_\mathrm{R}$ is given by: $\bar{s}_R = s_R/k_R^0 = k_R^0 R_\mathrm{T}/k_R^0 = R_\mathrm{T} \in (10^{-9}, 10^{-7})$ M. The estimated value of $\bar{R}_\mathrm{T}$ at this state (without miRNA regulation) is given by $\bar{R}_\mathrm{T} = R_\mathrm{T}/\bar{s}_R = R_\mathrm{T}/R_\mathrm{T} = 1$.

For simplicity, we dropped the bars in Eq 1.8.2 and we rewrote the system defined in Eq 1.8.1 as follows

$$\dot{R}_\mathrm{T} = 1 - (R + 2a_1C_1 + a_2C_2) \quad (1.8.3a)$$

$$\dot{r}_\mathrm{T} = \mu - \gamma(r + 2b_1C_1 + 2b_2C_2) \quad (1.8.3b)$$

$$0 = \kappa_1 Rr - C_1 \quad (1.8.3c)$$

$$0 = \kappa_2 C_1 r - C_2 \quad (1.8.3d)$$

$$0 = R + 2C_1 + C_2 - R_\mathrm{T} \quad (1.8.3e)$$

$$0 = r + 2C_1 + 2C_2 - r_\mathrm{T}. \quad (1.8.3f)$$

Here, $\kappa_1 = \bar{s}_R/K_1$ and $\kappa_2 = \bar{s}_R/K_2$. These parameters are essentially scaled association constants of mRNA-miRNA binding. Based on the estimated range of $\bar{s}_R$ mentioned earlier, and the estimated dissociation constant of mRNA-miRNA binding in the picomolar range (Wee *et al*, 2012), the approximate range of κ_1 and κ_2 is given by $\kappa_1, \kappa_2 = \bar{s}_R/K \in (10^{-9}\mathrm{M}/10^{-11}\mathrm{M}, 10^{-7}\mathrm{M}/10^{-12}\mathrm{M}) = (10^2, 10^5)$.

By eliminating R and r with the conservation relations, the system described in Eq 1.8.3 can be simplified to a four-variable system as follows

$$\dot{R}_\mathrm{T} = 1 - (R_\mathrm{T} - 2C_1 - C_2) - 2a_1C_1 - a_2C_2 \quad (1.8.4a)$$

$$\dot{r}_\mathrm{T} = \mu - \gamma(r_\mathrm{T} - 2C_1 - 2C_2) - 2\gamma b_1C_1 - 2\gamma b_2C_2 \quad (1.8.4b)$$

$$0 = \kappa_1(R_\mathrm{T} - 2C_1 - C_2)(r_\mathrm{T} - 2C_1 - 2C_2) - C_1 \quad (1.8.4c)$$

$$0 = \kappa_2 C_1(r_\mathrm{T} - 2C_1 - 2C_2) - C_2. \quad (1.8.4d)$$

Eq 1.8.4 is useful for performing numerical simulations to capture the system's dynamics, and for stability analysis. However, keeping the variables R_T and r_T makes it difficult to analyze the number of steady states of the system. In addition, the physical constraint $R, r \in \mathbb{R}_{0+}$ must be considered separately in addition to the domains of the four variables. Therefore, we also considered another four-variable system equivalent to Eq 1.8.4 describing the steady state of Eq 1.8.4 by keeping R and r instead of R_T and r_T

$$\dot{R}_\mathrm{T} = 0 \quad \Leftrightarrow \quad 0 = 1 - (R + 2a_1C_1 + a_2C_2) \quad (1.8.5a)$$

$$\dot{r}_\mathrm{T} = 0 \quad \Leftrightarrow \quad 0 = \mu - \gamma(r + 2b_1C_1 + 2b_2C_2) \quad (1.8.5b)$$

$$0 = \kappa_1 Rr - C_1 \quad (1.8.5c)$$

$$0 = \kappa_2 C_1 r - C_2. \quad (1.8.5d)$$

Under the tQSSA, replacing the ODEs for R_{T} and r_{T} with those for R and r will result in inaccuracy of the dynamics, but Eq 1.8.5 accurately describes the steady states of the system. In addition, the condition $R, r, C_1, C_2 \in \mathbb{R}_{0+}$ will directly give $R_{\mathrm{T}}, r_{\mathrm{T}} \in \mathbb{R}_{0+}$, so there is no additional constraint that needs to be considered.

1.8.2 Analysis of the number of steady states

The goal of this section is to find the parameter region in which the system described in Eq 1.8.4 has three steady states in $\mathbb{R}_{0+}$, which are necessary for bistability.

Theorem 1 Suppose $a_1, a_2, b_1, b_2, \kappa_1, \kappa_2 \in \mathbb{R}_{0+}$, and $\kappa_1 = \kappa_2 = \kappa \gg a_1/b_2$, $\kappa \gg 1/b_1$. There exists an η ($\eta \coloneqq \mu/\gamma$, $\eta \in \mathbb{R}_{+}$) such that Eq 1.8.4 has three real nonnegative equilibria if and only if

$$\frac{a_1}{b_1} < \frac{a_2}{2b_2}. \tag{1.8.6}$$

Justification of the assumptions The condition $\kappa_1 = \kappa_2 = \kappa \gg a_1/b_2$ and $\kappa \gg b_1$ means that the formation of the C_1 and C_2 are much more favored than their dissociations. The estimated range of κ is $(10^2, 10^5)$ (1.8.1). It was shown that the fold-changes of degradation rate constants of mRNA and mRNA upon their binding, described with a_1, a_2, b_1 and b_2, were estimated to be less than one order of magnitude (de la Mata *et al.*, 2015; Eichhorn *et al*, 2014). We therefore assume that these relationships are justified at least for a significant number of biological systems. In our numerical experiments presented in the next section, we relaxed this assumption and considered a wide range of values for κ (1.8.3).

Proof We first solve the Eq 1.8.5c for C_1 , and we obtain $C_1 = \kappa_1 R r$. Substituting C_1 with $\kappa_1 R r$ Eq 1.8.5d, and solving the equation for C_2 yields $C_2 = \kappa_1 \kappa_2 R r^2$. We take the simple assumption $\kappa_1 = \kappa_2 = \kappa$ (no cooperativity in binding), so that $C_2 = \kappa^2 R r^2$. We then eliminate C_1 and C_2 in Eq 1.8.5b, and solve it for R, and we obtain

$$R = \frac{\eta - r}{2\kappa r(b_1 + 2\kappa b_2 r)}, \quad \text{where } \eta = \frac{\mu}{\gamma} = \frac{s_r k_R^0}{s_R k_r^0}. \tag{1.8.7}$$

We then eliminate R and C_1 and C_2 in Eq 1.8.5a, and we obtain

$$0 = \kappa^2 a_2 r^3 + (-\kappa^2 a_2 \eta + 2\kappa^2 b_2 + 2\kappa a_1) r^2 + (-2\kappa a_1 \eta + 2\kappa b_1 + 1) r - \eta. \tag{1.8.8}$$

It follows from assumptions $\kappa \gg a_1/b_2$ and $\kappa \gg 1/b_1$ that $2\kappa^2 b_2 + 2\kappa a_1 \cong 2\kappa^2 b_2$, and $2\kappa b_1 + 1 \cong 2\kappa b_1$. Eq 1.8.8 can therefore be approximated by

$$0 = \kappa^2 a_2 r^3 + (-\kappa^2 a_2 \eta + 2\kappa^2 b_2) r^2 + (-2\kappa a_1 \eta + 2\kappa b_1) r - \eta. \tag{1.8.9}$$

We next use an algebraic geometry approach to find the conditions under which Eq 1.8.9 has three real positive equilibrium points (Siegal-Gaskins *et al*, 2015).

We first define the right-hand side of Eq 1.8.9 as $P(x)$, i.e.

$$P(r) = \kappa^2 a_2 r^3 + (-\kappa^2 a_2 \eta + 2\kappa^2 b_2) r^2 + (-2\kappa a_1 \eta + 2\kappa b_1) r - \eta. \tag{1.8.10}$$

We then construct the Sturm sequence for $P(x)$, i.e. a set of polynomials defined as

$$P_0 = P,$$

$$P_1 = P_0{}',$$

$$P_2 = -\text{rem}(P_1, P_0),$$

$$P_3 = -\text{rem}(P_2, P_1). \quad (1.8.11)$$

Here, $\text{rem}(P_{i-1}, P_i)$ is the remainder of the polynomial long division of P_i by P_{i-1}. It follows from Sturm's theorem that $P(r)$ has three real roots in interval $(0, \infty)$ if and only if $V(0) - V(\infty) = 3$ where V is number of sign variations in the sequence Eq 1.8.11. The sequence has four elements, therefore $V(0) \leq 3$, and since $V(\infty) \geq 0$, $P(r)$ has three real positive roots if and only if the sequence Eq 1.8.11 satisfies $V(0) = 3$ and $V(\infty) = 0$.

We calculate the Sturm sequence as follows

$$P_0 = \kappa^2 a_2 r^3 - (a_2\eta - 2b_2)\kappa^2 r^2 - 2(a_1\eta - b_1)\kappa r - \eta$$

$$P_1 = 3\kappa^2 a_2 r^2 - 2(a_2\eta - 2b_2)\kappa^2 r - 2\kappa(a_1\eta - b_1)$$

$$P_2 = 2(2\kappa(a_2\eta - 2b_2)^2 + 6a_2(a_1\eta - b_1))\kappa^5 r + 2a_2(a_1\eta - b_1)(a_2\eta - 2b_2)\kappa^5 + 9\kappa^4 a_2{}^2\eta$$

$$P_3 = 36a_2{}^3(a_2\eta - 2b_2)^2\big((a_1\eta - b_1)^2 - \eta(a_2\eta - 2b_2)\big)\kappa^{12} + O(\kappa^{11}). \quad (1.8.12)$$

Since $\lim_{r\to\infty} P_0(r) = \infty$ and $P_0(0) = -\eta < 0$, the only combination of signs in the sequence that allow three real positive roots is the following

	P_0	P_1	P_2	P_3
$r = 0$	-	+	-	+
$r \to \infty$	+	+	+	+

We next look for the conditions in terms of the parameters that satisfy all signs shown above by enumerating the remaining six inequalities with respect to P_1, P_2 and P_3.

For P_1, it follows from Eq 1.8.12 that $\lim_{r\to\infty} P_1(r) = \infty > 0$. In addition, $P_1(0) > 0$ if and only if

$$a_1\eta - b_1 < 0. \quad (1.8.13)$$

For P_2, it follows from Eq 1.8.12 and the assumption $\kappa \gg 1/b_1$ that

$$P_2(0) = 2a_2(a_1\eta - b_1)(a_2\eta - 2b_2)\kappa^5 + 9\kappa^4 a_2{}^2\eta$$

$$\cong 2a_2(a_1\eta - b_1)(a_2\eta - 2b_2)\kappa^5. \quad (1.8.14)$$

Similarly, $\lim_{r\to\infty} P_2(r) = \infty > 0$. It follows from Eq 1.8.12 and Eq 1.8.13 that $P_2(0) < 0$ if and only if

$$a_2\eta - 2b_2 > 0. \quad (1.8.15)$$

There exists an η ($\eta \in \mathbb{R}_+$) such that both $a_1\eta - b_1 < 0$ (Eq 1.8.13) and $a_2\eta - 2b_2 > 0$ (Eq 1.8.15) are satisfied if and only if Eq 1.8.6 is satisfied. In fact, $\eta \in (2b_2/a_2,\ b_1/a_1)$ when the two inequalities are satisfied.

P_3 does not depend on r, so $\lim_{r\to\infty} P_3(r) = P_3(0)$. It follows from Eq 1.8.12 and the assumption about κ that

$$\text{sgn}(P_3) = \text{sgn}\big((a_1\eta - b_1)^2 - \eta(a_2\eta - 2b_2)\big). \quad (1.8.16)$$

When $2b_2/a_2 = \eta$,

$$(a_1\eta - b_1)^2 - \eta(a_2\eta - 2b_2) = (a_1\eta - b_1)^2 > 0. \quad (1.8.17)$$

Therefore, there exists an $\eta^* \in (2b_2/a_2,\ b_1/a_1)$, such that for all $\eta \in (2b_2/a_2,\ \eta^*)$, $P_3 > 0$ if and only if Eq 1.8.6 is satisfied. Eq 1.8.6 is therefore the necessary and sufficient condition for Eq 1.8.5, and equivalently Eq 1.8.4, to have three real positive equilibrium points under the assumption $\kappa_1 = \kappa_2 = \kappa \gg a_1/b_2$, $\kappa \gg 1/b_1$, and $a_1, b_1, a_2, b_2 \in \mathbb{R}_{0+}$.

We next exclude the possibility that one of the four variables in Eq 1.8.5 can be zero in the solutions. Since $\mu \in \mathbb{R}_+$, $r = 0$ is not a solution to Eq 1.8.9. It follows from Eq 1.8.5c and Eq 1.8.5d that

$$R = 0 \Leftrightarrow C_1 = 0 \Leftrightarrow C_2 = 0, \quad (1.8.18)$$

and this does not satisfy Eq 1.8.5a. Therefore, when Eq 1.8.4 is at steady state, $r, R, C_1, C_2 \neq 0$. □

We next show that if Eq 1.8.6 is satisfied, the system described in Eq 1.8.4 switches from one equilibrium point, to three equilibrium point, then back to one equilibrium point when η continuously increases from zero to a number greater than b_1/a_1.

If $0 < \eta < 2b_2/a_2 < b_1/a_1$, the signs of Sturm sequences of $P(r)$ are $\{-,+,+,+\}$ and $\{+,+,+,+\}$ for $r = 0$ and $r \to \infty$, respectively. Therefore $V(0) - V(\infty) = 1$, i.e. Eq 1.8.5 has only one solution. In addition, if $\eta > b_1/a_1 > 2b_2/a_2$, the signs of Sturm sequences of $P(r)$ are $\{-,-,+,U\}$ and $\{+,+,+,U\}$ for $r = 0$ and $r \to \infty$, respectively, where U is an undetermined sign. Therefore $V(0) - V(\infty) = 1$, i.e. Eq 1.8.5 has only one solution.

1.8.3 Numerical experiments for bistable switches

Theorem 1 and the conclusion stated above may offer a simple framework for obtaining bistable switches with Eq 1.8.4, but they do not provide information about stability of the system Eq 1.8.4. Typical bistable switches require that in the parameter region with three steady states, two of the steady states are stable (stable nodes) and the remaining one is unstable (saddle point). In this scenario, changes in parameters allows the system to switch between three-steady-state region and one-steady state region via saddle-node bifurcations. However, we cannot make these conclusions based on the analysis described in 1.8.2. Although linear stability analysis under a limiting condition ($\kappa \to \infty$) provided some insights into stability (not shown), we were not able to obtain conclusive results about the stability for each of the three steady states. This implies that bifurcations other than saddle-node are possible with Eq 1.8.4. Here, we present numerical experiments that serve at least four purposes: 1) they validate conclusions of Theorem 1; 2) they show stability of each steady state in systems with various parameter sets; 3) they show the biochemical interpretations of off-state and on-state of the system and 4) they show the performance of Eq 1.8.6 in predicting the bistability of the system Eq 1.8.4 when the assumption about association constant κ is relaxed.

With the system described in Eq 1.8.4, we first chose a large association constant κ ($\kappa = 10^5$, i.e. the upper bound of our estimate in biological systems) that would satisfy the assumption of Theorem 1. We assumed that the basal degradation rate constant of miRNA is equal to that of the mRNA ($\gamma = 1$). For the relative degradation rate constants a_1, b_1, a_2, b_2, we selected 10^4 sets of values from uniformly distributed random numbers over the interval $(1/8, 16)$. These bounds were estimated from the altered stability of mRNA induced by miRNA binding, as well as the altered stability of miRNA induced by mRNA binding (de la Mata *et al.*, 2015; Eichhorn *et al.*, 2014). For each of the 10^4 parameter sets, we performed one-parameter

numerical bifurcation analysis with $\eta = 0$ as the starting point of control parameter. Unsurprisingly, letting $\eta = 0$ gave rise to a stable steady state where $r_T = 0$ and $R_T > 0$ with each parameter set. We define a system as a bistable switch if it has two saddle-node bifurcation points at two distinct values of η, which bound a region containing two stable nodes and one saddle point.

To compare the analytical prediction of bistability with Eq 1.8.6 with the results from the numerical experiments, we treated Eq 1.8.6 as a 'predictor' of bistable systems, and the numerically revealed bistable systems as 'true' bistable systems. This notion may be counterintuitive because analytical solutions are often used to evaluate numerical methods. In our case, the analytical conclusion serves as a guide to conduct numerical experiments and to derive intuitions of the feedback in this system, but the analysis involves a key biological assumption that needs to be evaluated and relaxed in biological applications. The detection capacity of the numerical method is limited by its precision, but the inaccuracy is neglected here. Among the 10^4 parameter sets (referred to as models), 3391 of them were predicted to bistable according to inequality Eq 1.8.6, and the remaining models were predicted to be monostable. 98.73% or 3348 of these predicted bistable systems were confirmed numerically, resulting in 43 false positives (numerically determined as monostable systems). Among the 6609 models that were predicted monostable according to the inequality Eq 1.8.6, 97.55% or 6527 of them were determined to be monostable numerically. The confusion matrix is shown in Table S2. Interestingly, 0.12% of the 10^4 parameter sets generated Hopf bifurcation point in addition to saddle-node bifurcation points, so that two of the three coexisting steady states are unstable. Since these systems are rare, we did not exclude them from the true bistable population, nor did we perform further analysis of these rare cases in this study.

Appendix Table S2. Confusion matrix for analytical prediction of bistable systems and numerical validations

	Predicted monostable	Predicted bistable
Monostable with numerical bifurcation	6527 (TP)	43 (FP)
bistable with numerical bifurcation	9 (FN)	3348 (TN)

TP: True positive. FP: False positive. FN: False negative. FN: True negative.

With visual inspection of the bifurcation diagrams of the numerically validated bistable systems (Figure 4C), we found that variables r_T, R_T, r, R and C_2 exhibited clear switches between on and off states with changes of η in most cases. This suggests that these bistable switches may be biochemical functional. Unsurprisingly, for r_T and R_T, the difference between on and off state is within one order of magnitude in most cases. In contrast, the on-off difference for r, R is typically greater than two orders of magnitude (Figure 4C). We found that similar fractions of parameter sets gave rise to bistable systems when we selected parameter values for a_1, b_1, a_2, b_2 from an interval other than $(1/8, 16)$, e.g. $(1, 16)$ or $(1/8, 1)$, and when we selected a_1, a_2 and b_1, b_2 from distinct intervals (Appendix Figure S1). Under these constraints of parameters, switch-like behaviors were also obtained with molecules such as r, R (Appendix Figure S2).

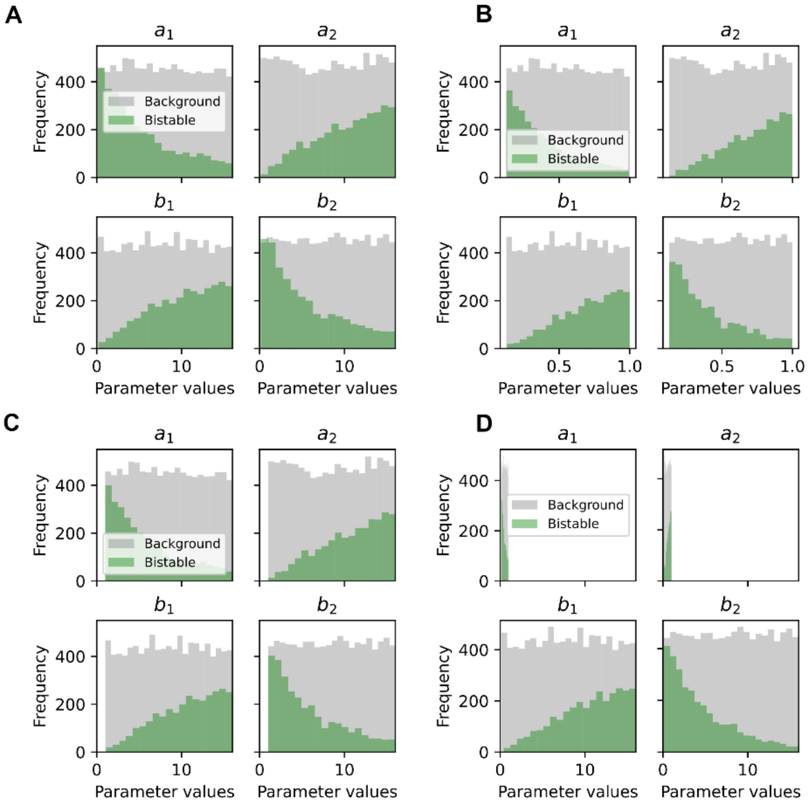

Appendix Figure S1. **Distributions of parameter values for bistable systems generated under the mmi-2 Model.** Distributions of randomly selected parameter values for four scaled degradation rate constants (gray) under the mmi-2 Model, as well as the subset that generated bistable systems (green). Each of the 10000 randomly selected parameter sets was tested for bistability by means of numerical bifurcation analysis under a basal parameter setting ($\gamma = 1, \kappa = 10^5$). Values of the four parameters were randomly drawn from uniform distributions over the intervals $(0.125, 16)$ (A), $(0.125, 1)$ (B), and $(1, 16)$ (C). In panel (D), values for a_1 and a_2 were randomly drawn from a uniform distribution over the interval $(0.125, 1)$, and values for b_1 and b_2 were randomly drawn from a uniform distribution over the interval $(1, 16)$.

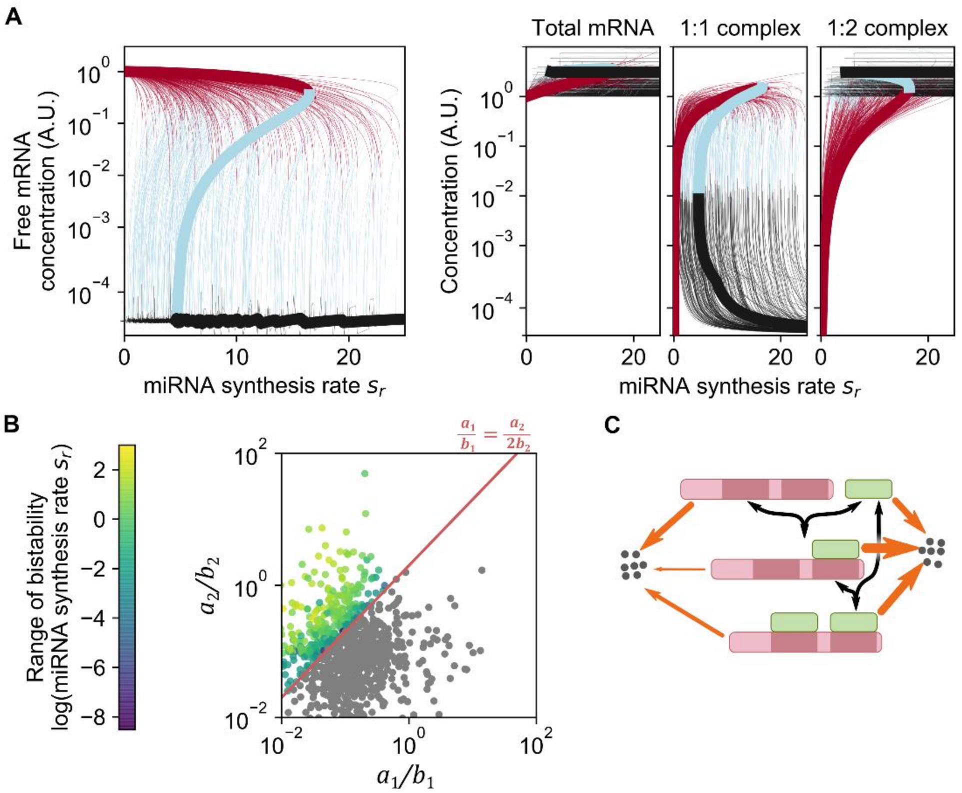

Appendix Figure S2. **Bistable systems generated from disproportionate degradation rate constants of miRNA and mRNA.** With the mmi-2 Model, we generated 10000 parameter sets with values for a_1 and a_2 randomly drawn from a uniform distribution over the interval $(0.125, 1)$, and for b_1 and b_2 randomly drawn from a uniform distribution over the interval $(1, 16)$. All other parameters, except the control parameter for bifurcation analysis and the scaled dissociation rate constant ($Kk_R^0/s_R = 10^{-5}$), were set to 1. (A) Bifurcation diagrams show steady states calculated for 3348 bistable systems out of 10000 parameter sets. Red curve: stable steady state with high amounts of free mRNA. Black curve: stable steady state with low amounts of free mRNA. Blue curve: unstable steady state. One representative system is highlighted with thick curves, and the other systems are depicted with thin curves. (B) Stability properties of the 10000 randomly generated systems. Monostable systems (gray dots) and bistable systems (purple-green dots) are shown in the dimensions of a_1/b_1 and a_2/b_2. The color gradient denotes the range of bistability in terms of the control parameter. Orange dot denotes the condition under which degradation of mRNA and miRNA is balanced in both complexes. Red line is the threshold for bistability predicted by analytical methods. (C) Network diagram of a mmi-2 Model with arrows representing bistability-enabling parameters described in A and B.

1.9 mRNA-miRNA model with three binding sites (mmi-3 Model)

The mRNA-miRNA model with three binding sites is simply an extension of the mmi-2 Model. We expanded Eq 1.8.4, a simplified system for the mmi-2 Model, by adding an equation for C_3, a partially double-stranded complex formed by three miRNA molecules bound to an mRNA molecule

$$\dot{R}_\mathrm{T} = 1 - (R_\mathrm{T} - 3C_1 - 3C_2 - C_3) - 3a_1C_1 - 3a_2C_2 - a_3C_3 \tag{1.9.1a}$$

$$\dot{r}_\mathrm{T} = \eta - (r_\mathrm{T} - 3C_1 - 6C_2 - 3C_3) - 3b_1C_1 - 6b_2C_2 - 3b_2C_2 \tag{1.9.1b}$$

$$0 = \kappa_1(R_\mathrm{T} - 3C_1 - 3C_2 - C_3)(r_\mathrm{T} - 3C_1 - 6C_2 - 3C_3) - C_1 \tag{1.9.1c}$$

$$0 = \kappa_2C_1(r_\mathrm{T} - 3C_1 - 6C_2 - 3C_3) - C_2 \tag{1.9.1d}$$

$$0 = \kappa_3C_2(r_\mathrm{T} - 3C_1 - 6C_2 - 3C_3) - C_3. \tag{1.9.1e}$$

We performed numerical experiments similar to those described in 1.8.3. Values for relative degradation rate constants $a_1, b_1, a_2, b_2, a_3, b_3$ were randomly selected from the interval $(1/8, 16)$. With numerical bifurcation analysis with respect to η using 10^4 parameter sets, we found that a high association constant ($\kappa = 10^5$) gave rise to 5081 bistable systems. The results with moderate association constant are presented in the next section.

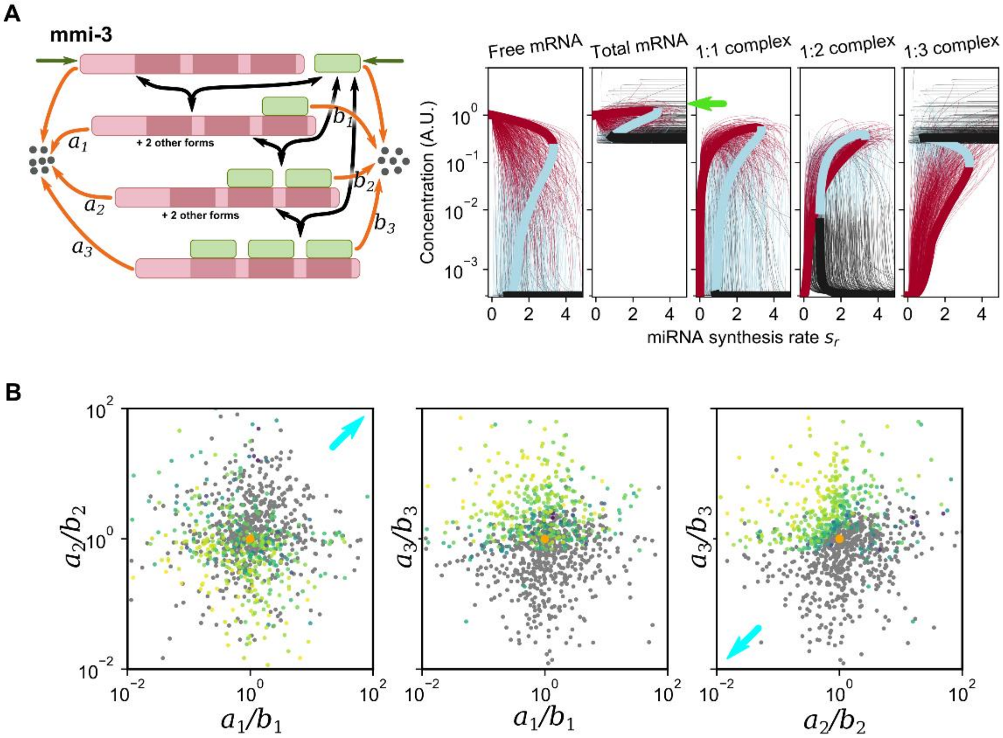

Appendix Figure S3. **Bifurcation analysis and parameter distributions of the mmi-3 Model**. (A) Left: mRNA-miRNA reaction network with three miRNA binding sites on the target mRNA (mmi-3 Model). Right: bifurcation analysis performed as described in Figure 4C. Of 10000 parameter sets, 5081 gave rise to bistable systems. Light green arrow denotes parameter sets in which mRNA degradation is not enhanced by complex formation. In these systems, a steady state is established in the presence of high amounts of miRNA but with total mRNA levels not less than 1. (B) Stability properties of 10000 randomly generated systems reflecting the mmi-3 Model based on the procedure described in the legend to Figure 4C. Monostable systems (gray dots) and bistable

systems (purple-green dots) are shown in the dimensions for a_1/b_1, a_2/b_2 and a_3/b_3. Color gradient denotes the range of bistability in terms of the control parameter. Orange dot denotes the condition under which degradation of mRNA and miRNA is balanced in the three complexes. Cyan arrows denote the parameter regions in which unidirectional asymmetry of the inhibitions only gave rise to monostable systems (left plot: complexes favor miRNA-mediated mRNA degradation; right plot: complexes favor mRNA-mediated miRNA degradation).

1.10 Performance of bistability predictions with reduced association constant

Under the basal parameter setting ($\kappa = 10^5$), we obtained 99.48% accuracy, 99.74% true positive rate, and 99.76% true negative rate using Eq 1.8.6 as a predictor for bistability with the mmi-2 Model. We next asked how this performance would change with the decrease of κ, i.e. relaxing the assumption of Theorem 1. We therefore repeated this numerical experiment with various values of κ (Appendix Figure S4A, pink, green, gold and purple). Furthermore, we found that the true positive rate was robust to the decrease of κ, whereas the true negative rate decreased as κ decreased (Appendix Figure S4A, green, and purple). Nonetheless, with a moderately high association constant ($\kappa = 10^2$) that is the lower bound of our estimate in biological systems (1.8.1), the accuracy and true negative rate were still higher than 90% (Appendix Figure S4A, gold, and purple), indicating that Eq 1.8.6 serves as a reasonable predictor of biological bistable systems governed by kinetics described in Eq 1.8.4. Furthermore, nearly 30% of the randomly selected parameter sets gave rise to bistable systems under this assumption of κ (Appendix Figure S4A, pink). Even with a low association constant ($\kappa = 1$), about three percent of the randomly generated parameter sets generated bistable systems (Appendix Figure S4A, pink), which were correctly predicted by Eq 1.8.6. These results suggest that the reaction network described in Eq 1.8.4 may be a widely used motif for generating bistable switches in biology. In addition, we found that restricting a_1, b_1, a_2, b_2 to $(1/8, 1)$ or $(1, 16)$ reduced the fractions of parameter sets that generated bistable systems, but the decrease was not dramatic (Appendix Figure S4, cyan and yellow).

For mmi-3 Model, a moderately high association constant ($\kappa = 10^2$) gave rise to 4022 bistable systems out of the 10^4 parameter sets (Appendix Figure S4A, blue and B). The distributions of individual parameters from bistable systems were wider than those from mmi-2 bistable systems (Appendix Figure S4B). These results show that with the increased number of binding sites, it is even more feasible for the system to achieve bistability with biologically plausible kinetic rate constants.

1.11 Estimate of realistic biological circuits described by mmi-2 and mmi-3 Models

To estimate how frequently the mRNA-miRNA reaction network motif represented by mmi-2 and mmi-3 models can be found in biological systems, we obtained a data set for predicted miRNA binding sites in human and mouse from TargetScan (Agarwal *et al*, 2015). To estimate the lower bound of the number of appearances, we counted the number of mRNA-miRNA pairs in which the target mRNA has two or more conserved binding sites for the cognate miRNA. To estimate the upper bound, we counted the number of miRNA binding site duplets or triplets (conserved and non-conserved) each of which share target mRNA and cognate miRNA. The numbers of predicted target gene, cognate miRNA and mRNA-miRNA circuit are listed in Appendix Table S3.

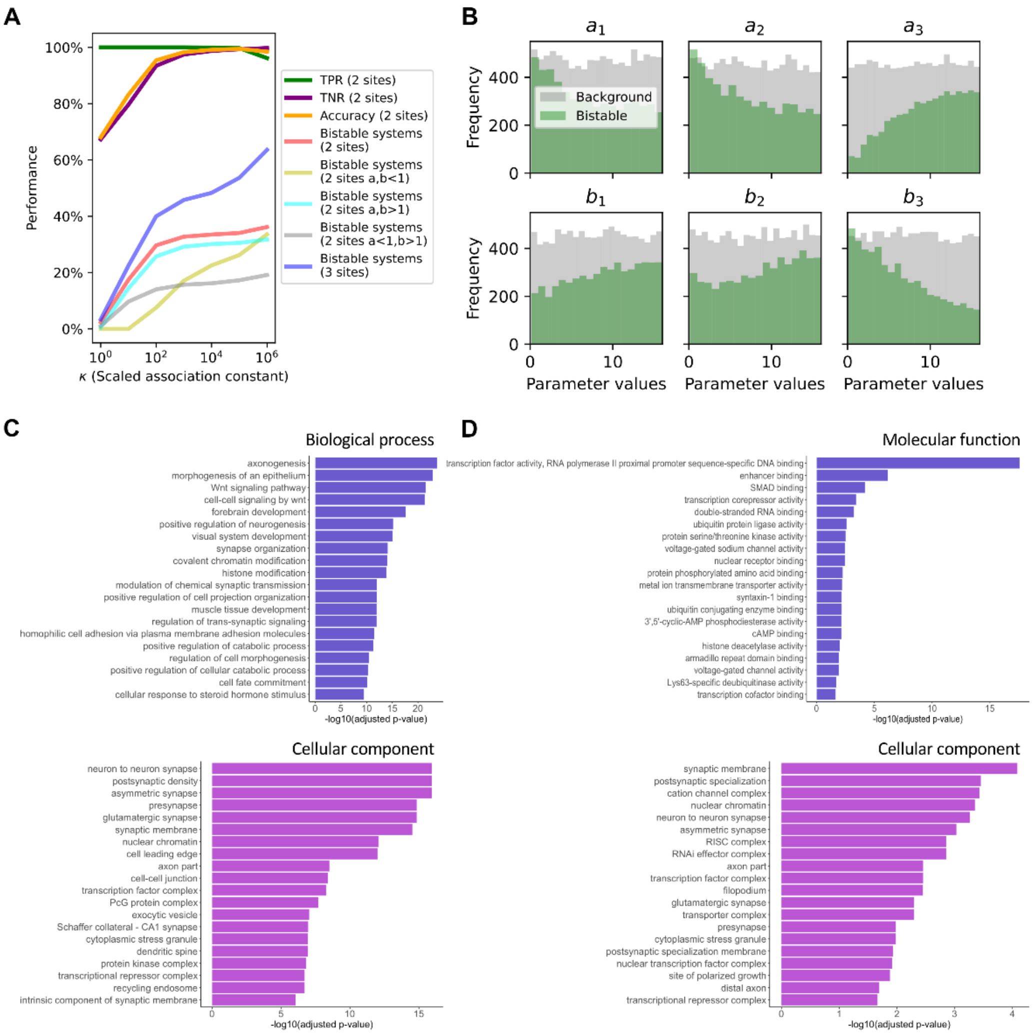

Appendix Figure S4. **Performance of analytical predictions for bistable systems under various models and distributions of parameters values for bistable systems generated with the mmi-3 Model. (**A) The three top lines of the graph show the performance (TPR: true positive rate; TNR: true negative rate; and accuracy) of Eq 1.8.6 in predicting the bistability of the mmi-2 model. Other lines show the fractions of bistable systems with various constraints of parameter sampling, model choice, and values of κ. (B**).** Distributions of randomly selected parameter values for six scaled degradation rate constants (gray) under the mmi-3 Model and the subset that generated bistable systems (green). Each of the 10000 randomly selected parameter sets was tested for bistability by numerical bifurcation analysis under a basal parameter setting ($\gamma = 1, \kappa = 10^5$). Values of the four parameters were randomly drawn from uniform distributions over the intervals $(0.125, 16)$. (C) Gene ontology enrichment analysis for miRNA targeted genes with 2 or more binding sites and (D) 3 or more binding sites. Bar plot shows the top 20 significant terms related to biological process or molecular function (top) and cellular component (bottom).

Appendix Table S3. Numbers of predicted mRNAs, miRNAs and circuits that can be described by the mmi-2 and -3 Models

Quantities predicted by TargetScan	Human	Mouse
Genes involved in ≥ 2 binding sites of any miRNA	3800 – 8561*	2738 – 6560
miRNA involved in ≥ 2 binding sites on any mRNA	291 – 309	275 – 303
Unique mRNA-miRNA pairs described by the mmi-2 Model	9571 – 45328	5896 – 27723
Distinct circuits described by the mmi-2 Model	13337 – 122885	7908 – 60345
Genes involved in ≥ 3 binding sites of any miRNA	804 – 5027	458 – 3225
miRNA involved in ≥ 3 binding sites on any mRNA	194 – 294	161 – 288
Distinct circuits described by the mmi-3 Model	3322 – 93049	1960 – 32314
Unique mRNA-miRNA pairs described by the mmi-3 Model	1250 – 16914	664 – 8631

* Lower bounds were obtained from conserved sites; upper bounds were obtained from all predicted sites.

1.12 Effects of a competitor mRNA on bistable switches governed by mmi-2 Model

In our mmi-2 and mmi-3 Models, we only considered one target for the miRNA. This is unlikely to be realistic because miRNAs usually have multiple target genes. We therefore tested the effects of a competitor mRNA on the ability for an mmi-2 Model to give rise to bistability. In addition to a miRNA-target pair (R and r), we considered a hypothetical competitor mRNA Rc that can bind to miRNA (r). Here, we focused our discussion on the ability of the system to have a clear bistable switch for the primary target mRNA R. First, we considered two scenarios (Rc has two binding sites and one binding site). If Rc has two binding sites and the triggered degradation rate constants satisfy Eq 1.2.1 ($2a_1/b_1 < a_2/b_2$), then the system is bistable for both R and Rc. This is an obvious scenario because the variable R in the original mmi-2 Model can be viewed as the total concentration of multiple targets with similar properties. We therefore focused on the situation where Rc cannot generate bistability by itself, and the triggered degradation rate constants follow the average experimentally estimated quantities ($2a_1 = a_2 = 2$, $b_1 = b_2 = 1$) (Grimson *et al.*, 2007). The degradation rate constants for R are assumed to be moderately cooperative ($3a_1 = a_2 = 3$), which gives rise to a small bistable region (Appendix Figure S5A, top left). We varied the transcription rate constants for Rc, as well as the relative dissociation constants of the primary target and the competitor mRNA, and performed bifurcation analysis for 15 cases (Appendix Figure S5A). As expected, when target-miRNA binding is 10,000 times stronger than the competitor-miRNA binding, the system showed a clear on-off bistable switch for R (Appendix Figure S5A, top row), and when target-miRNA binding is 10,000 times weaker than the competitor-miRNA binding, the bistable switch disappeared (Appendix Figure S5A, bottom row). Interestingly, in most other cases, the clear on-off bistable switches were retained (Appendix Figure S5A), including a situation where the target-miRNA binding is 100 times weaker than the competitor-miRNA binding. The only parameter region that resulted in the disappearance of the bistable switches is when the competitor concentration is at least comparable to the target concentration and the target-miRNA binding is 100 times stronger than the competitor-miRNA binding. We found that the overall effects of a single-binding-site competitor were similar to the double-binding-site competitor that is not bistability enabling (Appendix Figure S5B). In conclusion, the competitor mRNA may have a negative impact on the bistable switch mechanism proposed in this study, but in a wide range of biologically plausible parameter values, the bistability was still retained. The effect depends on at least four factors: 1) the relative abundance of the primary target and competitors; 2) the number of binding sites on the competitor mRNA; 3) the altered degradation rate constants upon binding of the competitor mRNA and

miRNA and 4) the dissociation constant of the binding between the primary target and miRNA, and that between the competitor mRNA and the same miRNA.

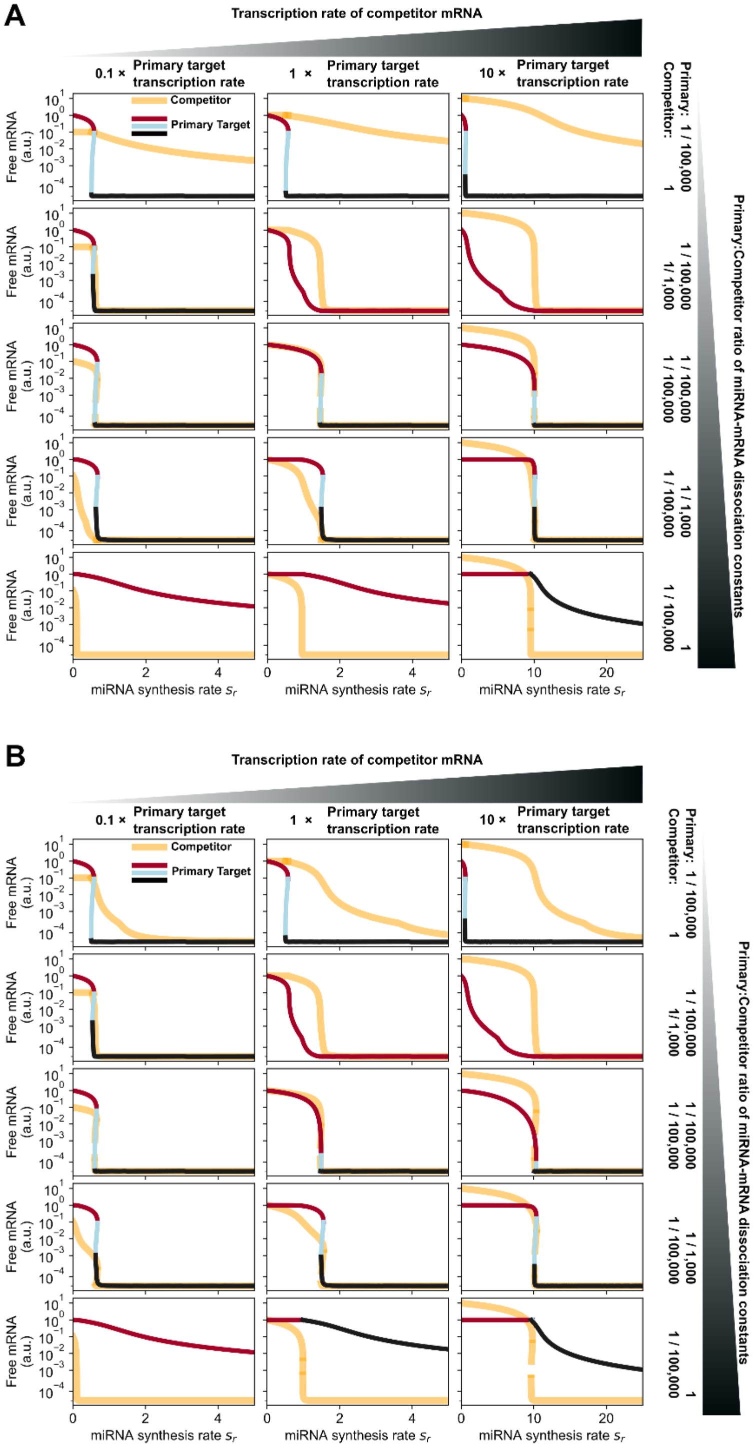

Appendix Figure S5. Bifurcation diagrams of the mmi-S Model in the presence of a competitor mRNA. The mmi-S Model was combined with a set of equations describing a

competitor mRNA Rc, which shares the same regulatory miRNA with R (a primary target considered in this study). Red curve: stable steady state with high amounts of free primary target mRNA. Black curve: stable steady state with low amounts of free primary target mRNA. A. The competitor mRNA has two binding sites. The degradation rate constants are additive, but not cooperative ($2a_1 = a_2 = 2$), which does not allow it to generate a bistable switch by itself (right). B. The competitor mRNA has one binding site. Light blue curve: unstable steady state of free primary target mRNA. No TDMD was considered for this competition model. Orange curve: steady state of free competitor mRNA (Solid: stable. Dashed: unstable). Scaled dissociation constants (Kk_R^0/s_R, Kk_{RC}^0/s_{RC}) were varied for plots from top to bottom with the indicated values. Transcription rate constants (s_{RC}) of the competitor mRNA were varied for plots from left to right with the indicated values. All other parameters, except the control parameter for bifurcation analysis, were set to 1.

1.13 mRNA-miRNA with noncanonical feedback and morphogen gradients (mmi-S Model)

The form of equation is identical to Tmi-UR model (Eq1.2.8, Eq 1.4.1 and Eq 1.4.2). The parameters were chosen based on the principle derived in 1.8 and 1.9 (mmi-2 and mmi-3 models), such that both Hoxa5-miR-27 axis and Hoxc8-miR-196 axis are bistable. The parameter values of this and other models are listed in Table S4 and Table S5.

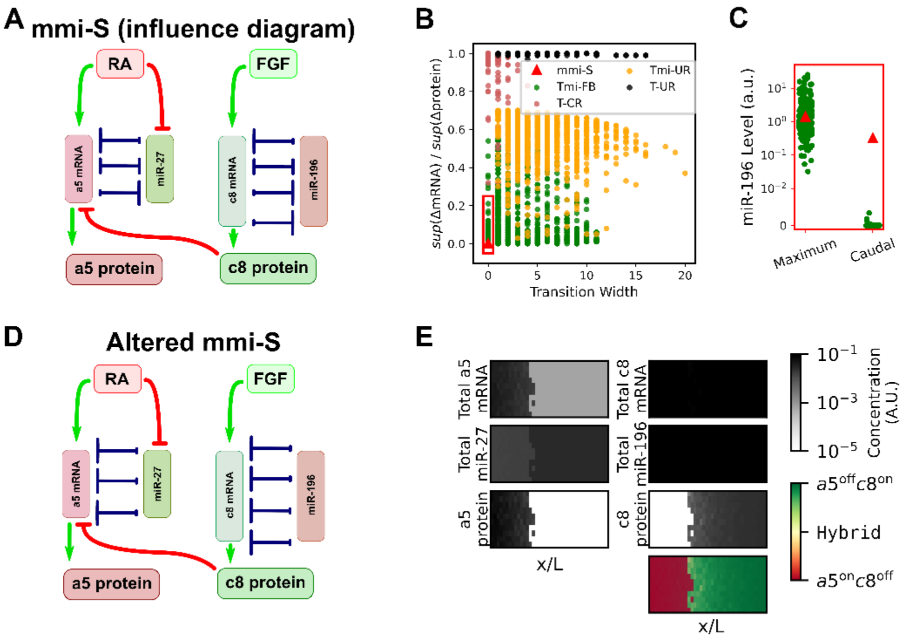

Appendix Figure S6. **Performance and perturbation of the mmi-S Model.** (A) Influence diagram of the feedback loops in the mmi-S Model (Figure 8A). (B) Performance of the mmi-S Model overlaid with that of four other models presented in Figure 4G. (C) Performance of the mmi-S Model overlaid with that of the Tmi-FB Model presented in Figure 4H. (D) Influence diagram of an altered mmi-S Model exhibiting unidirectional regulation of mRNA by miRNA,

which does not allow feedback formation. Only intracellular parts of the models are shown. (E) Tissue domain patterning at the final time point for the altered mmi-S Models.

1.14 List of parameter values and ranges for random sampling

Appendix Table S4. List of parameter values and ranges for random sampling

Parameter	Description	Value in T-CR	Value in T-UR	Value in Tmi-UR	Value in Tmi-FB	Sampling Range *	Value in mmi-S
s_5^0	Basal production rate constant of *Hoxa5* mRNA	0	0	0.15	0.15	μ=0.01, σ=1.5	0.06
s_5	Regulated production rate constant of *Hoxa5* mRNA	1	1	1	1	μ=10, σ=1.5	2
K_{5A}	Threshold of *Hoxa5* activation by RA	0.7	0.7	0.98	0.48	μ=1, σ=0.5	7.2
n_{5A}	Response nonlinearity of *Hoxa5* activation by RA	6	60 **	6	6	6	2
K_{58}	Threshold of *Hoxa5* inhibition by Hoxc8	0.05	0.05	0.1	0.05	μ=0.1, σ=1.5	0.006
n_{58}	Response nonlinearity of *Hoxa5* inhibition by Hoxc8	6	20 **	40 **	6	6	2
s_8^0	Basal production rate constant of *Hoxc8* mRNA	0	0	0.05	0	μ=0.01, σ=1.5	0
s_8	Regulated production rate constant of *Hoxc8* mRNA	1	1	1	1	μ=10, σ=1.5	1.2
K_{8F}	Threshold of *Hoxc8* activation by FGF	0.3	0.75	0.95	2.3	μ=1, σ=0.5	0.95
n_{8F}	Response nonlinearity of *Hoxc8* activation by FGF	6	60 **	40 **	6	6	6
K_{85}	Threshold of *Hoxc8* inhibition by Hoxa5	NA	0.18	NA	NA	μ=0.1, σ=1.5	NA
n_{85}	Response nonlinearity of *Hoxc8* inhibition by Hoxa5	NA	6	NA	NA	6	NA
l_5^0	Translation rate constant of free *Hoxa5* mRNA	1	1	1	1	μ=2, σ=1	1.8
l_8^0	Translation rate constant of free *Hoxc8* mRNA	0.7	1	1	5	μ=2, σ=1	3.9
k_5	Degradation rate constant of free *Hoxa5* mRNA	1	1	1	1	1	1
k_8	Degradation rate constant of free *Hoxc8* mRNA	1	1	1	1	1	1
k_5^i	Degradation rate constant of *Hoxa5* mRNA in complex C_5^i	NA	NA	1	3	μ=2, σ=1.5	Table S4
k_8^i	Degradation rate constant of *Hoxc8* mRNA in complex C_8^i	NA	NA	1	1	μ=2, σ=1.5	Table S4
l_5^i	Translation rate constant of complex C_5^i	NA	NA	0	0	0	0
l_8^i	Translation rate constant of complex C_8^i	NA	NA	0	0	0	0
K_{2A}	Threshold of miR-27 inhibition by RA	NA	NA	0.9	0.9	μ=1, σ=0.5	3.2
n_{2A}	Response nonlinearity of miR-27 inhibition by RA	NA	NA	6	6	6	6
k_2	Regulated production rate constant of miR-27	NA	NA	1	1	μ=1, σ=1.5	0.16
k_9	Regulated production rate constant of miR-196	NA	NA	1	1	μ=1, σ=1.5	0.8
k_2^i	Degradation rate constant of miR-27 in complex C_5^i	NA	NA	1	1	μ=1, σ=1.5	Table S4
k_9^i	Degradation rate constant of miR-196 in complex C_8^i	NA	NA	1	1	μ=1, σ=1.5	Table S4
κ_5^i	Association constant of complex C_5^i formation	1000	1000	1000	1000	1000	1000
κ_8^i	Association constant of complex C_8^i formation	1000	1000	1000	1000	1000	1000
K_{25}	Threshold of miR-27 inhibition by Hoxa5	NA	NA	NA	0.1	μ=0.1, σ=1.5	NA
n_{25}	Response nonlinearity of miR-27 inhibition by Hoxa5	NA	NA	NA	6	6	NA
K_{98}	Threshold of miR-196 inhibition by Hoxc8	NA	NA	NA	0.02	μ=0.1, σ=1.5	NA
n_{98}	Response nonlinearity of miR-196 inhibition by Hoxc8	NA	NA	NA	6	6	NA

* Parameter values were assumed to be constants, or randomly drawn from log-normal distributions with the indicated μ and σ. All models use the same distributions, where the parameters are applicable.

** Extremely high nonlinearity was assumed to estimate the upper bound of the performance.

Appendix Table S5. Additional parameter values for mmi-S Model

Parameter	Description	Value in mmi-S Model
k_5^1	Degradation rate constant of *Hoxa5* mRNA in complex C_5^1	0.3
k_5^2	Degradation rate constant of *Hoxa5* mRNA in complex C_5^2	0.53
k_5^3	Degradation rate constant of *Hoxa5* mRNA in complex C_5^3	1.53
k_8^1	Degradation rate constant of *Hoxc8* mRNA in complex C_8^1	1
k_8^2	Degradation rate constant of *Hoxc8* mRNA in complex C_8^2	1
k_8^3	Degradation rate constant of *Hoxc8* mRNA in complex C_8^3	1.5
k_8^4	Degradation rate constant of *Hoxc8* mRNA in complex C_8^4	2.66
k_2^1	Degradation rate constant of miR-27 in complex C_5^1	1
k_2^2	Degradation rate constant of miR-27 in complex C_5^2	1
k_2^3	Degradation rate constant of miR-27 in complex C_5^3	0.6
k_9^1	Degradation rate constant of miR-196 in complex C_8^1	2.7
k_9^2	Degradation rate constant of miR-196 in complex C_8^2	2.1
k_9^3	Degradation rate constant of miR-196 in complex C_8^3	0.75
k_9^4	Degradation rate constant of miR-196 in complex C_8^4	0.12

* The bifurcation diagrams shown in Figure 6H were produced under the same parameter settings as the mmi-S Model, except for the following parameters: $l_5^0 = 0.03$, $l_5^1 = 0.5$, $K_{5A} = 0.7$, $n_{5A} = 6$, $K_{2A} = 0.6$. The mmi-S Model under with these parameter values produced results similar to those shown in Figure 7.

As described in 1.6, model parameters were first randomly chosen from defined distributions which are consistent across all models. Model evaluations are based on statistics obtained from the parameter sampling. To show representative simulation results, we chose one parameter set for each model and adjusted some parameters manually for illustration purposes. The range of parameter sampling and the parameter values of representative models are listed in Table S4 and Table S5.

2. Additional Information of Resources

2.1 List of key reagents and resources

Appendix Table S6. List of key reagents and resources

Reagent type (species) or resource	Designation	Source or reference	Identifiers	Additional information
Antibody	Goat anti-Isl1	Neuromics	Cat# 39.4D5, RRID: AB_2314682	ICC (1:1000)
Antibody	Mouse monoclonal anti-Isl1(2)	DSHB	Cat# 39.4D5, RRID: AB_2314683	ICC (1:1000)
Antibody	Rabbit polyclonal anti-Hoxa5	Jeremy Dasen (NYU)		ICC (1:16000)
Antibody	Guinea pig polyclonal pig anti-Hoxa5	Jun-An Chen (Academia Sinica)	RRID: AB_2744661	ICC (1:20000)
Antibody	Mouse monoclonal anti-Hoxc8	DSHB	Cat# PCRP-HOXC8-1D3, RRID: AB_2618723	ICC (1:1000)
Antibody	Rabbit polyclonal anti-Hoxc8	Sigma-Aldrich	Cat# HPA028911, RRID: AB_10602236	ICC (1:5000)
Antibody	Sheep polyclonal anti-GFP	AbD Serotec	Cat# 4745-1051, RRID: AB_619712	ICC (1:1000)
Antibody	Guinea pig polyclonal anti-Hb9	Hynek Wichterle (Columbia University)		ICC (1:1000)
Antibody	Mouse monoclonal anti-Hb9	DSHB	Cat# 81.5C10, RRID: AB_2145209	ICC (1:200)
Cell line	Mouse: *Hb9::GFP* (*Mnx1::GFP*) ESCs	(Wichterle *et al*, 2002)		Dr. Hynek Wichterle (Columbia University)
Mouse strain	*Hoxc8::Cre*	(Carroll & Capecchi, 2015)		Dr. Mario Capecchi (University of Utah)
Mouse strain	*ROSA26-loxp-STOP-loxp-tdTomato*	(Madisen *et al*, 2010)	The Jackson Lab **(**Stock No. 007914**)**	Dr. Hong-Kui Zeng (Allen Institute)
Cell line	Mouse: *iHoxa5-V5* ESCs	this study		
Cell line	Mouse: *iHoxc8-V5* ESCs	(Li *et al.*, 2017)		
Cell line	Mouse: *imiR-ScrmSP* ESCs	(Li *et al.*, 2017)		

Cell line	Mouse: *imiR-27 SP* ESCs	(Li *et al.*, 2017)		
Cell line	Mouse: *iGFP* ESCs	(Li *et al.*, 2017)		
Cell line	Mouse: *imiR-196a OE* ESCs	this study		
Cell line	Mouse: *imiR-27b OE* ESCs	this study		
Mouse strain	*Hb9::GFP*	(Wichterle *et al.*, 2002)		
Mouse strain	*miR-23a~27a~24-2*$^{+/-}$*; miR-23b~27b~24-1*$^{+/-}$	(Li *et al.*, 2017)		
Mouse strain	*miR-196a1*$^{+/-}$ *; miR-196a2*$^{-/-}$ *; miR-196b*$^{-/-}$	(Wong *et al.*, 2015)		
In situ probe	mmu-Hoxa5	(Li *et al.*, 2017)		
In situ probe	mmu-Hoxc8	(Li *et al.*, 2017)		
In situ probe	mmu-miR-27b	QIAGEN	MIMAT0000126	
Commercial assay or kit	Neural Tissue Dissociation Kit (P)	Miltenyi Biotec	130-092-628	
Commercial assay or kit	Chromium Single Cell 3' Reagent Kits v3.1	10X Genomics	PN-1000121	
Software, algorithm	10X Cell Ranger v3.1.0	10X Genomics	RRID: SCR_017344	
Software, algorithm	Seurat v2.3.4	(Butler *et al*, 2018; Stuart *et al*, 2019)	RRID: SCR_016341	
Software, algorithm	MetaMorph Microscopy Automation and Image Analysis Software		RRID: SCR_002368	
Software, algorithm	Differential Equation Solver: Differentialequations.jl 6.14.0	(Rackauckas & Nie, 2017)		
Software, algorithm	Numerical Bifurcation Analysis: Tellurium 2.1.5, AUTO	(Choi *et al*, 2018; Doedel, 1981)		

2.2 Primers for 3´ UTR mutants

Appendix Table S7. Primer sequences for 3´UTR mutants

Gene	Site	Forward Primer	Reverse Primer
Hoxc8	Site 1	CTCTATGATGGAGTCAGATAGT TGCAGCTCTGC	CTGACTCCATCATAGAGTTGTGAG TGTCATTCTATTCTC
Hoxc8	Site 2	AAATCGATGGATTCCATCCCAT AGTCACTTGGG	ATGGAATCCATCGATTTCAGAGAC CATTGCCAGTTC
Hoxc8	Site 3	AACATTGATGGATAAGGGAACC TGCAATAATCTTGGG	CCCTTATCCATCAATGTTAAAGAT TTATTATCAGTATATTATTTTCATG ATTATGG
Hoxa5	Site 1	TCCTTGACACTTGAAGCCCTGTT CTCGTTGCCC	GCTTCAAGTGTCAAGGAACACAA GGGGAGT TTCAG
Hoxa5	Site 2	GCTTGGAGAGTGTCATTTCGTC ACAGAGCCACTAGCC	CGAAATGACACTCTCCAAGCGGTG TGTCCC
Hoxa5	Site 3	AGCAATGACACTTCAGCTTTTTT TTTTTTTTGTATTTGTTTTTAAG G	GCTGAAGTGTCATTGCTTAAACAG CCAGACTTGG

2.3 Primers for genotyping

Appendix Table S8. Primers for genotyping

Gene	Forward Primer	Reverse Primer
GFP	CCCTGAAGTTCATCTGCACCAC	TTCTCGTTGGGGTCTTTGCTC
Cre	TGATGGACATGTTCAGGGATC	CAGCCACCAGCTTGCATGA
Ai14 WT	AAGGGAGCTGCAGTGGAGTA	CCGAAAATCTGTGGGAAGTC
Ai14 Mutant	CTGTTCCTGTACGGCATGG	GGCATTAAAGCAGCGTATCC
miR-27a WT	GGGAATGCTTCTTCCCTCTT	CACGACTTTGCTGTGGACCT
miR-27a Del	GGGAATGCTTCTTCCCTCTT	CTATCTGCTTTGGGGAACCA
miR-27b WT	CTCTGTGCTATGCCTCAGCTTAT	CCCCATCTCACCTTCTCTTCAG
miR-27b Del	CTCTGTGCTATGCCTCAGCTTAT	TCAGAAAGGCTCTACAGACAAGG

2.4 Sequence for miRNA sponge

miR-27b sponge (8 repeats, ccgg as spacer)

5’

GCAGAACTTCGGACTGTGAAccggGCAGAACTTCGGACTGTGAAccggGCAGAACTTCGGACTG
TGAAccggGCAGAACTTCGGACTGTGAAccggGCAGAACTTCGGACTGTGAAccggGCAGAACTT
CGGACTGTGAAccggGCAGAACTTCGGACTGTGAAccggGCAGAACTTCGGACTGTGAA

mir-scramble sponge (8 repeats, ccgg as spacer)

5’
TTCACAATGCGTTATCGGATGTccggTTCACAATGCGTTATCGGATGTccggTTCACAATGCGTT
ATCGGATGTccggTTCACAATGCGTTATCGGATGTccggTTCACAATGCGTTATCGGATGTccggT
TCACAATGCGTTATCGGATGTccggTTCACAATGCGTTATCGGATGTccggTTCACAATGCGTTA
TCGGATGT

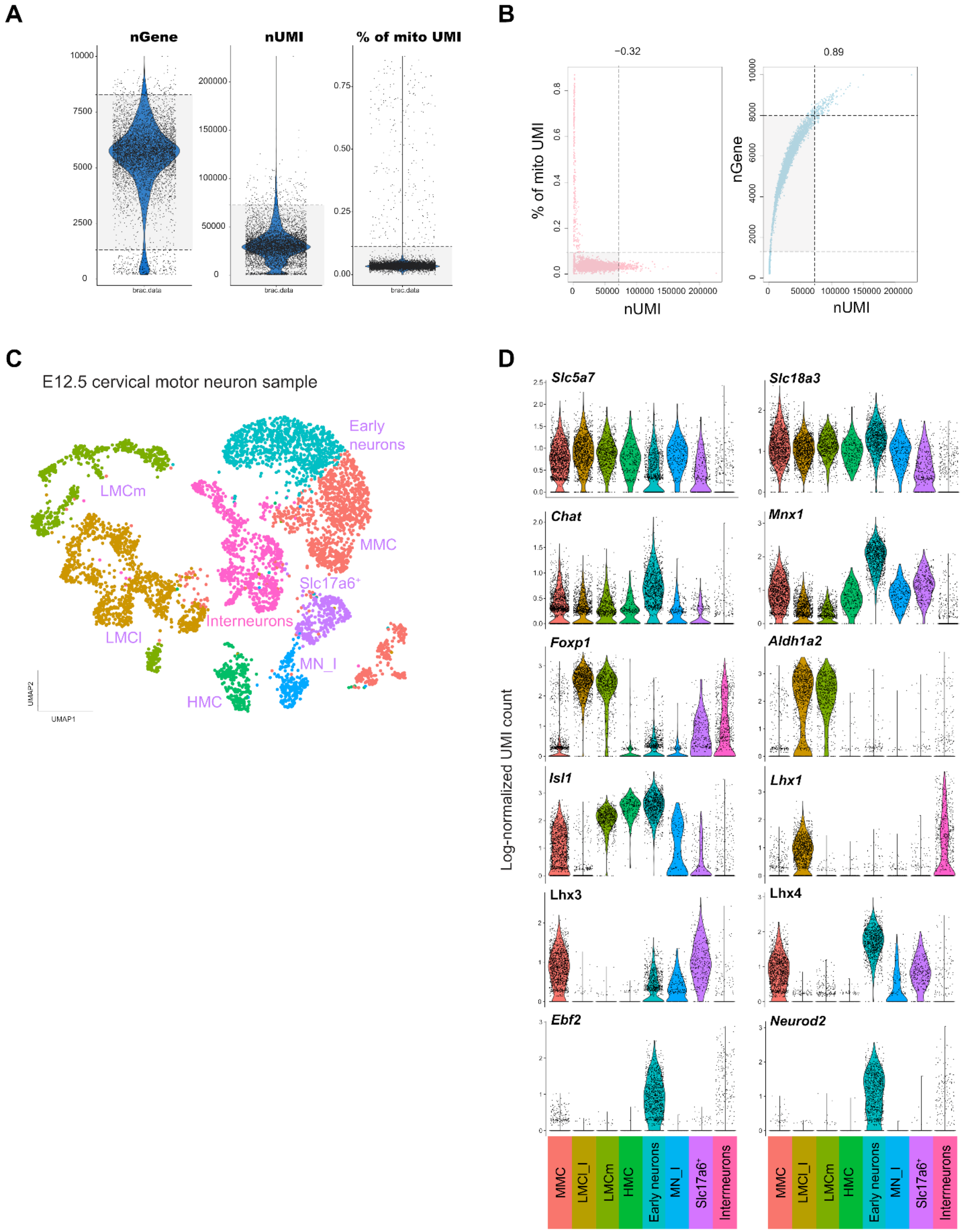

Appendix Figure S7. **Quality assessment and clustering analysis of collected E12.5 spinal motor neurons.** (A) Violin plot reflecting the number of expressed genes (nGene), unique

molecular identifier counts (nUMI), and percentage of UMIs for mitochondrial genes for the collected single cells. (B) Scatter plot depicting the relationship between the features in (A) for quality filtering. Cells expressing fewer than 1300 genes and with more than 10% of UMI counts related to mitochondrial genes were excluded from downstream analyses. Dashed lines indicate the filtering cutoffs. (C) UMAP plot of 5581 cells upon quality filtering. Each cell is color-coded based on clustering results using a Louvain-Jaccard algorithm. Identities are assigned according to the expression of known MN markers as presented in (D). The eleven identified major clusters mainly comprised brachial LMC and MMC MNs based on prior experience (see Materials & Methods and SI Text for details).

Appendix References

Agarwal V, Bell GW, Nam J-W, Bartel DP (2015) Predicting effective microRNA target sites in mammalian mRNAs. *eLife* 4: e05005

Borghans JAM, De Boer RJ, Segel LA (1996) Extending the quasi-steady state approximation by changing variables. *Bull Math Biol* 58: 43-63

Butler A, Hoffman P, Smibert P, Papalexi E, Satija R (2018) Integrating single-cell transcriptomic data across different conditions, technologies, and species. *Nat Biotechnol* 36: 411-420

Carroll LS, Capecchi MR (2015) Hoxc8 initiates an ectopic mammary program by regulating Fgf10 and Tbx3 expression and Wnt/beta-catenin signaling. *Development* 142: 4056-4067

Choi K, Medley JK, König M, Stocking K, Smith L, Gu S, Sauro HM (2018) Tellurium: an extensible python-based modeling environment for systems and synthetic biology. *Biosystems* 171: 74-79

Ciliberto A, Capuani F, Tyson JJ (2007) Modeling networks of coupled enzymatic reactions using the total quasi-steady state approximation. *PLoS Comput Biol* 3: e45

Dasen JS, Tice BC, Brenner-Morton S, Jessell TM (2005) A Hox regulatory network establishes motor neuron pool identity and target-muscle connectivity. *Cell* 123: 477-491

de la Mata M, Gaidatzis D, Vitanescu M, Stadler MB, Wentzel C, Scheiffele P, Filipowicz W, Großhans H (2015) Potent degradation of neuronal miRNAs induced by highly complementary targets. *EMBO Rep* 16: 500-511

Doedel EJ (1981) AUTO: A program for the automatic bifurcation analysis of autonomous systems. *Congr Numer* 30: 265-284

Eichhorn SW, Guo H, McGeary SE, Rodriguez-Mias RA, Shin C, Baek D, Hsu S-h, Ghoshal K, Villén J, Bartel DP (2014) mRNA destabilization is the dominant effect of mammalian microRNAs by the time substantial repression ensues. *Mol Cell* 56: 104-115

Ensini M, Tsuchida TN, Belting H-G, Jessell TM (1998) The control of rostrocaudal pattern in the developing spinal cord: specification of motor neuron subtype identity is initiated by signals from paraxial mesoderm. *Development* 125: 969-982

Feinberg M (2019) *Foundations of Chemical Reaction Network Theory*. Springer International Publishing

Ghini F, Rubolino C, Climent M, Simeone I, Marzi MJ, Nicassio F (2018) Endogenous transcripts control miRNA levels and activity in mammalian cells by target-directed miRNA degradation. *Nat Commun* 9: 3119

Grimson A, Farh KK-H, Johnston WK, Garrett-Engele P, Lim LP, Bartel DP (2007) MicroRNA targeting specificity in mammals: determinants beyond seed pairing. *Mol Cell* 27: 91-105

Kloosterman WP, Wienholds E, de Bruijn E, Kauppinen S, Plasterk RHA (2006) In situ detection of miRNAs in animal embryos using LNA-modified oligonucleotide probes. *Nat Methods* 3: 27-29

Lahtvee P-J, Sánchez BJ, Smialowska A, Kasvandik S, Elsemman IE, Gatto F, Nielsen J (2017) Absolute quantification of protein and mRNA abundances demonstrate variability in gene-specific translation efficiency in yeast. *Cell systems* 4: 495-504

Li C-J, Hong T, Tung Y-T, Yen Y-P, Hsu H-C, Lu Y-L, Chang M, Nie Q, Chen J-A (2017) MicroRNA filters Hox temporal transcription noise to confer boundary formation in the spinal cord. *Nat Commun* 8: 14685

Lu M, Jolly MK, Gomoto R, Huang B, Onuchic J, Ben-Jacob E (2013) Tristability in cancer-associated microRNA-TF chimera toggle switch. *The journal of physical chemistry B* 117: 13164-13174

Madisen L, Zwingman TA, Sunkin SM, Oh SW, Zariwala HA, Gu H, Ng LL, Palmiter RD, Hawrylycz MJ, Jones AR *et al* (2010) A robust and high-throughput Cre reporting and characterization system for the whole mouse brain. *Nat Neurosci* 13: 133-140

Mazzoni EO, Mahony S, Peljto M, Patel T, Thornton SR, McCuine S, Reeder C, Boyer LA, Young RA, Gifford DK (2013) Saltatory remodeling of Hox chromatin in response to rostrocaudal patterning signals. *Nat Neurosci* 16: 1191-1198

Philippidou P, Dasen JS (2013) Hox genes: choreographers in neural development, architects of circuit organization. *Neuron* 80: 12-34

Rackauckas C, Nie Q (2017) Differentialequations. jl–a performant and feature-rich ecosystem for solving differential equations in julia. *Journal of Open Research Software* 5

Riba A, Bosia C, El Baroudi M, Ollino L, Caselle M (2014) A combination of transcriptional and microRNA regulation improves the stability of the relative concentrations of target genes. *PLoS Comput Biol* 10: e1003490

Siegal-Gaskins D, Franco E, Zhou T, Murray RM (2015) An analytical approach to bistable biological circuit discrimination using real algebraic geometry. *Journal of The Royal Society Interface* 12: 20150288

Sosnik J, Zheng L, Rackauckas CV, Digman M, Gratton E, Nie Q, Schilling TF (2016) Noise modulation in retinoic acid signaling sharpens segmental boundaries of gene expression in the embryonic zebrafish hindbrain. *eLife* 5: e14034

Stuart T, Butler A, Hoffman P, Hafemeister C, Papalexi E, Mauck Iii WM, Hao Y, Stoeckius M, Smibert P, Satija R (2019) Comprehensive integration of single-cell data. *Cell* 177: 1888-1902

Tian XJ, Zhang H, Zhang J, Xing J (2016) Reciprocal regulation between mRNA and microRNA enables a bistable switch that directs cell fate decisions. *FEBS Lett* 590: 3443-3455

Wee LM, Flores-Jasso CF, Salomon WE, Zamore PD (2012) Argonaute divides its RNA guide into domains with distinct functions and RNA-binding properties. *Cell* 151: 1055-1067

Wichterle H, Lieberam I, Porter JA, Jessell TM (2002) Directed differentiation of embryonic stem cells into motor neurons. *Cell* 110: 385-397

Wong SFL, Agarwal V, Mansfield JH, Denans N, Schwartz MG, Prosser HM, Pourquié O, Bartel DP, Tabin CJ, McGlinn E (2015) Independent regulation of vertebral number and vertebral identity by microRNA-196 paralogs. *Proc Natl Acad Sci USA* 112: E4884-E4893
