# Supplementary material for: MicroRNA governs bistable cell differentiation and lineage segregation via a noncanonical feedback
Source: Mol Syst Biol. 2021 Apr 23;17(4):e9945. doi: 10.15252/msb.20209945 (PMC8062999; doi:10.15252/msb.20209945)
Supplement: Supplementary file 2 — Expanded View Figures PDF [file MSB-17-e9945-s009.pdf]

Expanded View Figures

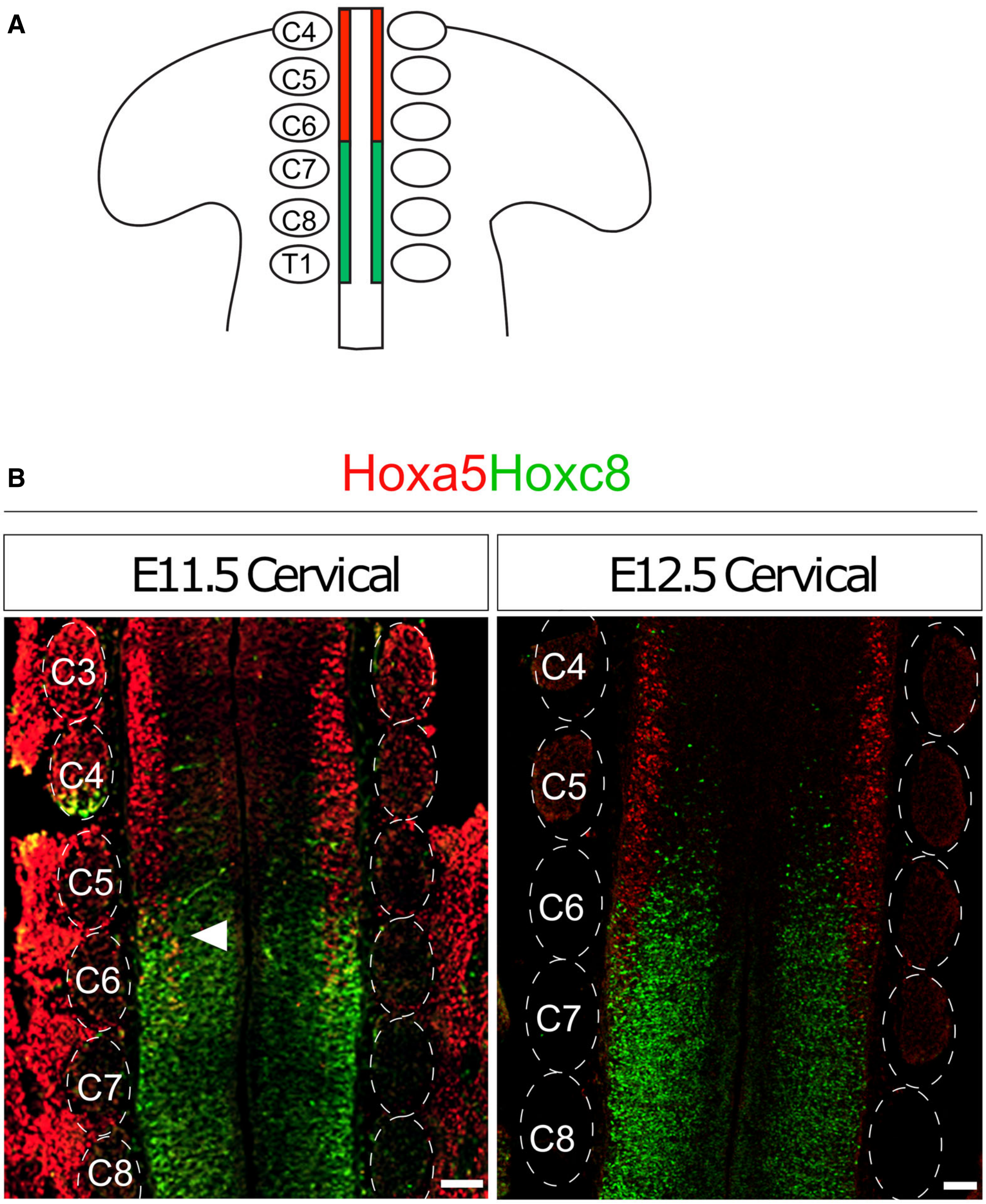

Figure EV1. Expression of Hoxa5 and Hoxc8 along the rostrocaudal axis of the spinal cord.

A Schematic illustration of the juxtaposed positioning of cervical segments in Figs 1 and EV1B. Red and green boxes represent a region of $Hoxa5^{on}$ cells and a region of $Hoxc8^{on}$ cells, respectively.

B Immunostainings of Hoxa5 and Hoxc8 reveal the lineage segregation and boundary sharpening process between E11.5 and E12.5. Longitudinal sections of the spinal cord in which dorsal root ganglia were preserved were used to determine positions of cervical segments. Arrowhead indicates co-expression of Hoxa5 and Hoxc8 proteins at E11.5. Scale bar represents 100 μm.

© 2021 The Authors

Figure EV2. Transcriptional cross-repression (T-CR) and transcriptional unilateral repression (T-UR) models.

A Schematic of the transcriptional cross-repression model (T-CR model).

B Presumptive simulated time-course of RA and FGF signaling at multiple locations along the rostrocaudal (RC) axis. A.U.: arbitrary unit.

C Simulation of the T-CR model. A grid of 10 × 40 cells was used to represent a segment of developing spinal cord where progenitor cells are influenced by competing FGF and RA concentrations. Heatmaps reflect final distributions of denoted molecules in the tissue domain. Bottom panel shows the distribution of the ratios between Hoxa5 and Hoxc8 protein levels.

D Steady-state levels of Hoxa5 and Hoxc8 proteins across RC domains. Error bar indicates 95% confidence interval (obtained with bootstrapping of 10 replicates) for each position receiving the same amount of morphogen. Transition width is defined as number of positions where one or more cells have equivocal lineage decision.

E Network diagram of the T-UR model. Unlike the T-CR model, regulation in this model is supported by experimental data. The T-UR model was simulated in the same way as for the T-CR model (panel A), including an assumed time-course of RA and FGF signaling at multiple locations along the rostrocaudal axis.

F Simulation of the T-UR model. A grid of 10 × 40 cells was used to represent a segment of developing spinal cord where progenitor cells are influenced by competing FGF and RA concentrations. Heatmaps show final distributions of denoted molecules in the tissue domain. Bottom panel shows the distribution of ratios between Hoxa5 and Hoxc8 protein levels.

G Steady-state levels of Hoxa5 and Hoxc8 proteins across RC domains. Error bar indicates 95% confidence interval (obtained with bootstrapping of 10 replicates) for each position receiving the same amount of morphogen.

H Bifurcation analysis with position as the control parameter. Solid curves denote stable steady states. Black line represents the steady state of the T-UR model. Dashed curves denote unstable steady states. Other lines represent the steady states of the T-CR model. Red: $Hoxa5^{on}Hoxc8^{off}$ state and green: $Hoxa5^{off}Hoxc8^{on}$ state. The identities of these states were determined by protein levels, which are equivalent to free mRNA levels.

I Distributions of transition widths from simulations with 10,000 parameter sets for each of the T-CR and T-UR models. Parameter sets with transition widths < 10 are shown.

J Network diagram of a system incorporating hypothetical positive feedbacks upstream of Hoxa5 and Hoxc8 via transcriptional control. Dashed lines represent hypothetical (unvalidated) regulations that may improve lineage segregation performance. Hoxa5 and Hoxc8 self-activation is also considered one type of hypothetical positive feedback.

© 2021 The Authors

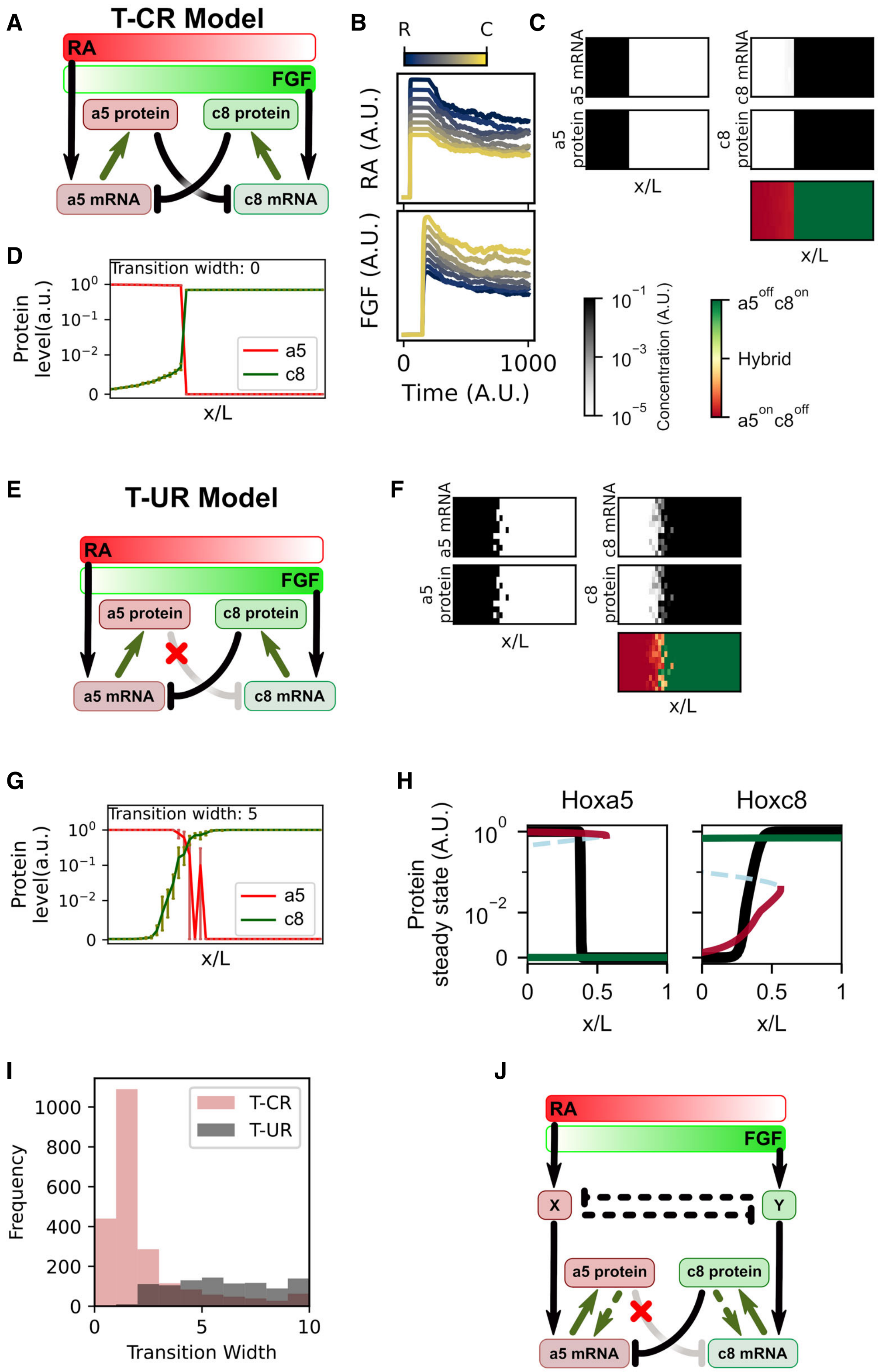
A
T-CR Model
RA
FGF
a5 protein
c8 protein
a5 mRNA
c8 mRNA
B
R
C
RA (A.U.)
FGF (A.U.)
0
1000
Time (A.U.)
C
a5 mRNA
a5 protein
c8 mRNA
c8 protein
x/L
x/L
Concentration (A.U.)
a5off c8on
Hybrid
a5on c8off
D
Transition width: 0
Protein level(a.u.)
a5
c8
x/L
E
T-UR Model
RA
FGF
a5 protein
c8 protein
a5 mRNA
c8 mRNA
F
a5 mRNA
a5 protein
c8 mRNA
c8 protein
x/L
x/L
G
Transition width: 5
Protein level(a.u.)
a5
c8
x/L
H
Hoxa5
Hoxc8
Protein steady state (A.U.)
x/L
x/L
I
Frequency
T-CR
T-UR
Transition Width
J
RA
FGF
X
Y
a5 protein
c8 protein
a5 mRNA
c8 mRNA

Figure EV2.

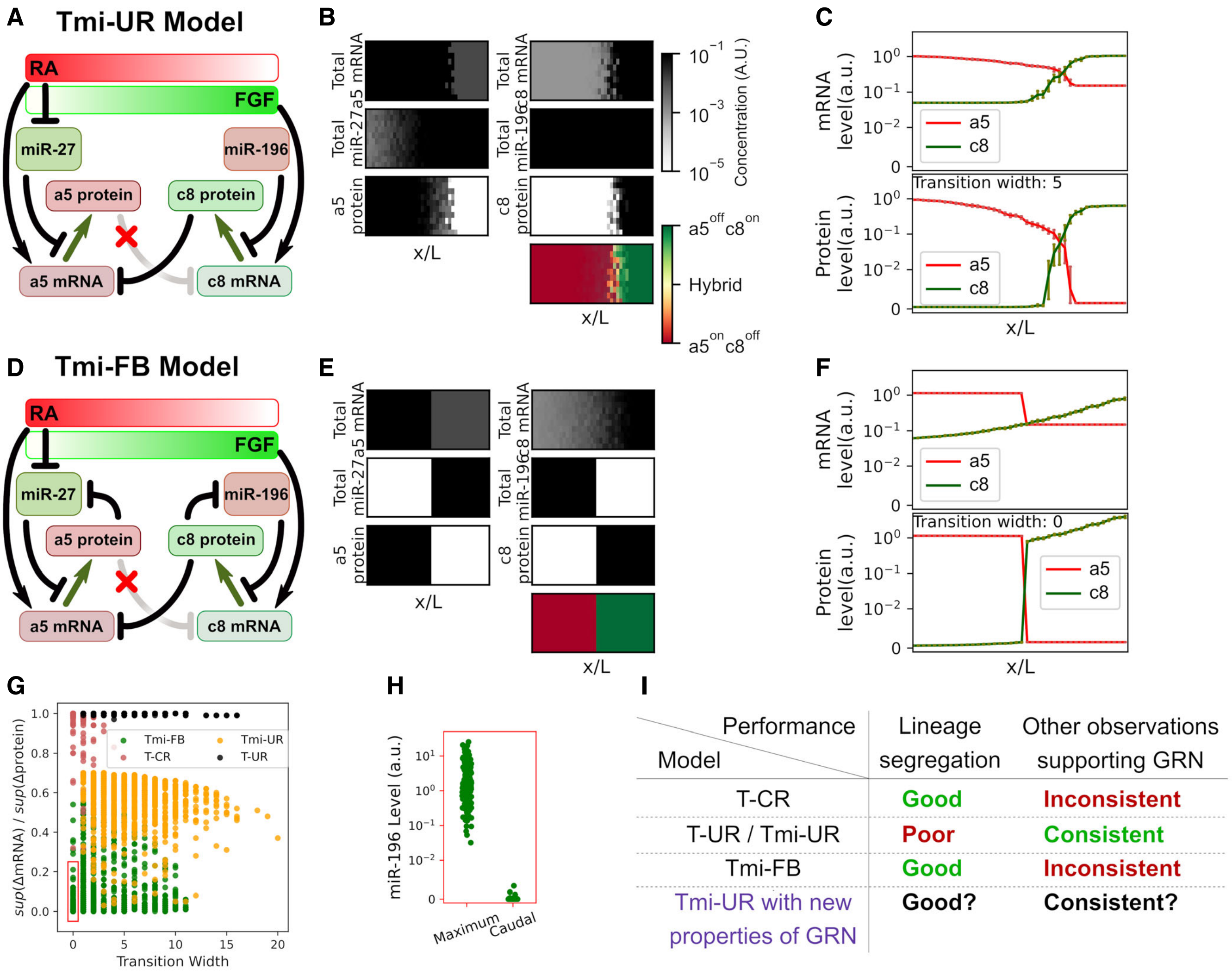

Figure EV3. Two alternative models of miRNA-mediated regulation.

- A Schematic of the model representing transcriptional unilateral repression with miRNA-mediated regulation (Tmi-UR model).
- B Simulation of the Tmi-UR model. A grid of 10 × 40 cells was used to represent a segment of developing spinal cord where progenitor cells are influenced by competing FGF and RA concentrations. Heatmaps show the final distributions of denoted molecules in the tissue domain. Bottom panel shows the distribution of ratios between Hoxa5 and Hoxc8 protein levels.
- C Steady-state levels of *Hoxa5* and *Hoxc8* mRNA (top) and proteins (bottom) across RC domains under Tmi-UR model. Error bar indicates 95% confidence interval for each position receiving the same amount of morphogen.
- D Schematic of the model representing transcriptional unilateral repression with miRNA-mediated feedback (Tmi-FB model). Hypothetical mRNA–miRNA feedback involving transcriptional repression of miRNA by target mRNA is assumed.
- E Simulation of the Tmi-FB model. A grid of 10 × 40 cells was used to represent a segment of developing spinal cord where progenitor cells are influenced by competing FGF and RA concentrations. Heatmaps show final distributions of denoted molecules in the tissue domain. Bottom panel shows the distribution of ratios between Hoxa5 and Hoxc8 protein levels. Color scales are the same as those in (B).
- F Steady-state levels of *Hoxa5* and *Hoxc8* mRNA (top) and proteins (bottom) across RC domains under Tmi-FB model. Error bar indicates 95% confidence interval (obtained with bootstrapping of 10 replicates) for each position receiving the same amount of morphogen.
- G Performance of 3000 top performing parameter sets from randomly generated values for each model (Appendix Table S4). y-coordinates are mRNA-to-protein ratios in terms of gradient steepness along the RC axis (segregation index, see Appendix Supplementary Methods for details). Red square indicates selected 190 sets (100% from Tmi-FB) for further analysis.
- H Quantifications of steady-state *miR-196* levels for models selected from (G). Caudal boundary level is compared to maximum level across the RC domain.
- I Summary of lineage decision performance and consistency with experimental data for four models.

 © 2021 The Authors

Figure EV4. Luciferase assay with 3′ UTR and Hoxa5 expression in response to RA in ESC differentiation.

A Predicted targeting sites for *miR-196* in the *Hoxc8* 3′ UTR (left panel) and for *miR-27* in the *Hoxa5* 3′ UTR (right panel), based on TargetScan.

B Left panel: Luciferase reporters were constructed with either a control *Hoxc8* 3′ UTR or the 3′ UTR sequence in which the individual or multiple potential target sites of *miR-196* were mutated (red). Right panel: Co-expression of a luciferase construct with *miR-196a* in HeLa cells silences a reporter carrying intact *miR-196* target sites, whereas *miR-196* fails to fully silence Mut1 (site#1 mutated), Mut2/3 (site#2 and site#3 mutated), and Mut1/2/3 (site#1, site#2, and site#3 mutated) luciferase constructs ($N = 3$ independent experiments, mean ± SD, *$P < 0.05$, **$P < 0.01$, Student's *t*-tests).

C Left panel: Luciferase reporters were constructed with either a control *Hoxa5* 3′ UTR or the 3′ UTR sequence in which the individual or multiple potential target sites of *miR-27* were mutated (red). Right panel: Co-expression of a luciferase construct with *miR-27b* in ES cells silences a luciferase reporter constructs carrying one or more mutated sites ($N = 3$ independent experiments, mean ± SD, *$P < 0.05$, **$P < 0.01$, Student's *t*-tests).

D Schematic illustration of the experiments in (E) and (F).

E Immunostainings of Hoxa5 and Hb9 in embryoid bodies with a concentration gradient of RA ranging from 100 nM to 1 μM.

F Quantification of Hoxa5^{on} cells from the Hb9^{on} population.

Data information: Scale bar in (D) represents 50 μm. Data in (F) represent mean ± SD, $N \geq 3$ EBs from three independent experiments.

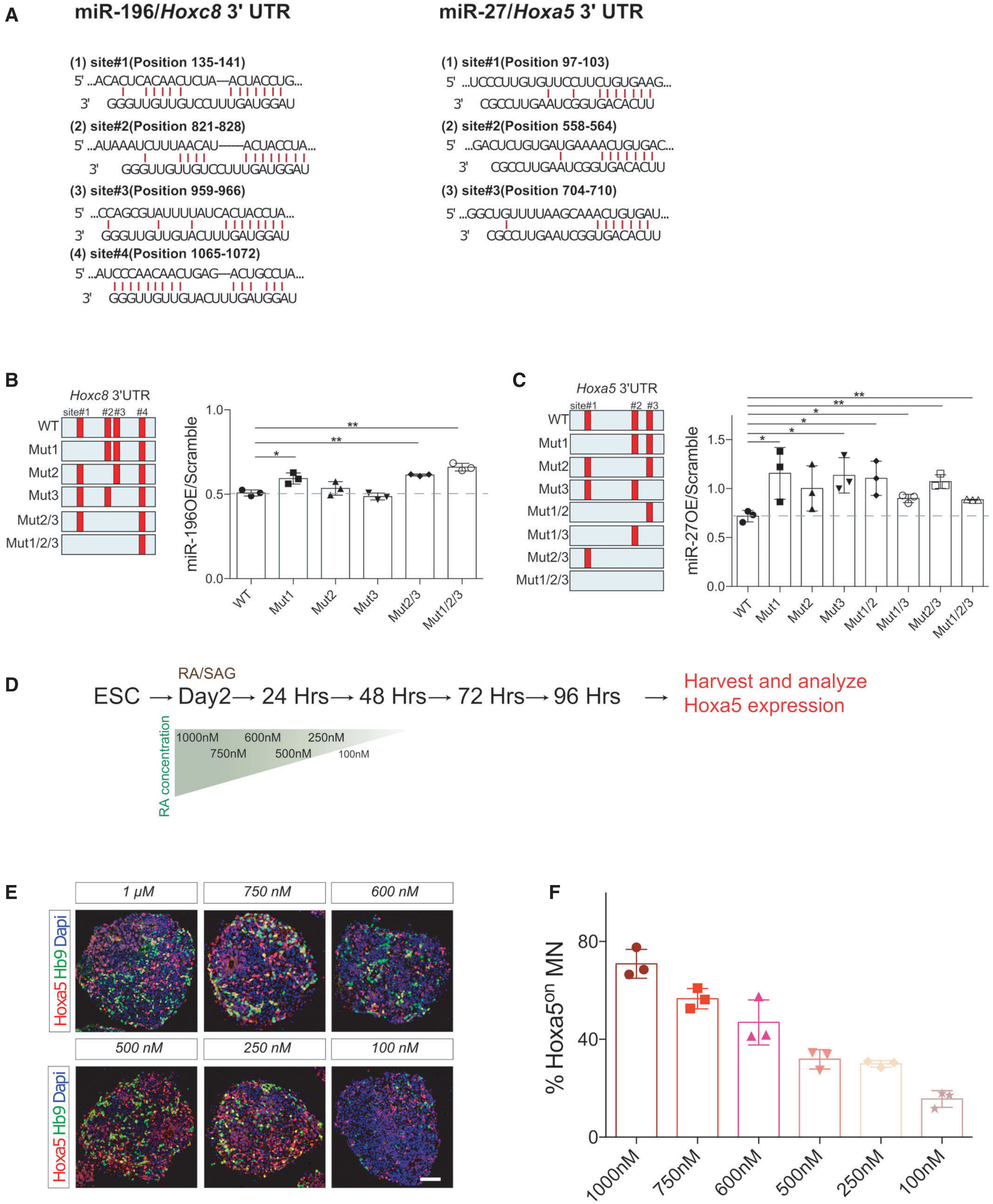

Figure EV4.

© 2021 The Authors

Figure EV5. Overexpression of *miR-27* and *miR-196* leads to the efficient repression of Hoxa5 and Hoxc8 in spinal MNs.

A, B Schematic illustrations of the generation of inducible ESC lines expressing primary miRNA sequences inserted into the GFP 3′ UTR. ESCs were differentiated under conditional MN differentiation conditions with doxycycline treatment on day 4 of differentiation.

C–F Expression of Hoxa5/Hoxc8 and Hb9/Isl1 in EBs from control (iGFP) and iMir-27b- or iMir-196a-overexpressing (OE) cells. Induction of *miR-27b* on day 4 of differentiation under RA/SAG conditions resulted in reduced Hoxa5 levels (D), whereas induction of *miR-196a* repressed Hoxc8 expression (F). Both conditions have no discernible effect on MN differentiation, as revealed by Hb9 or Isl1 expression (D and F). Pink scale bar in (C) and (E) represents 50 μm. Data in (D) and (F) represent mean ± SD, $N \geq 4$ EBs from three independent experiments, $^{*}P < 0.01$, Student's *t*-tests.

© 2021 The Authors

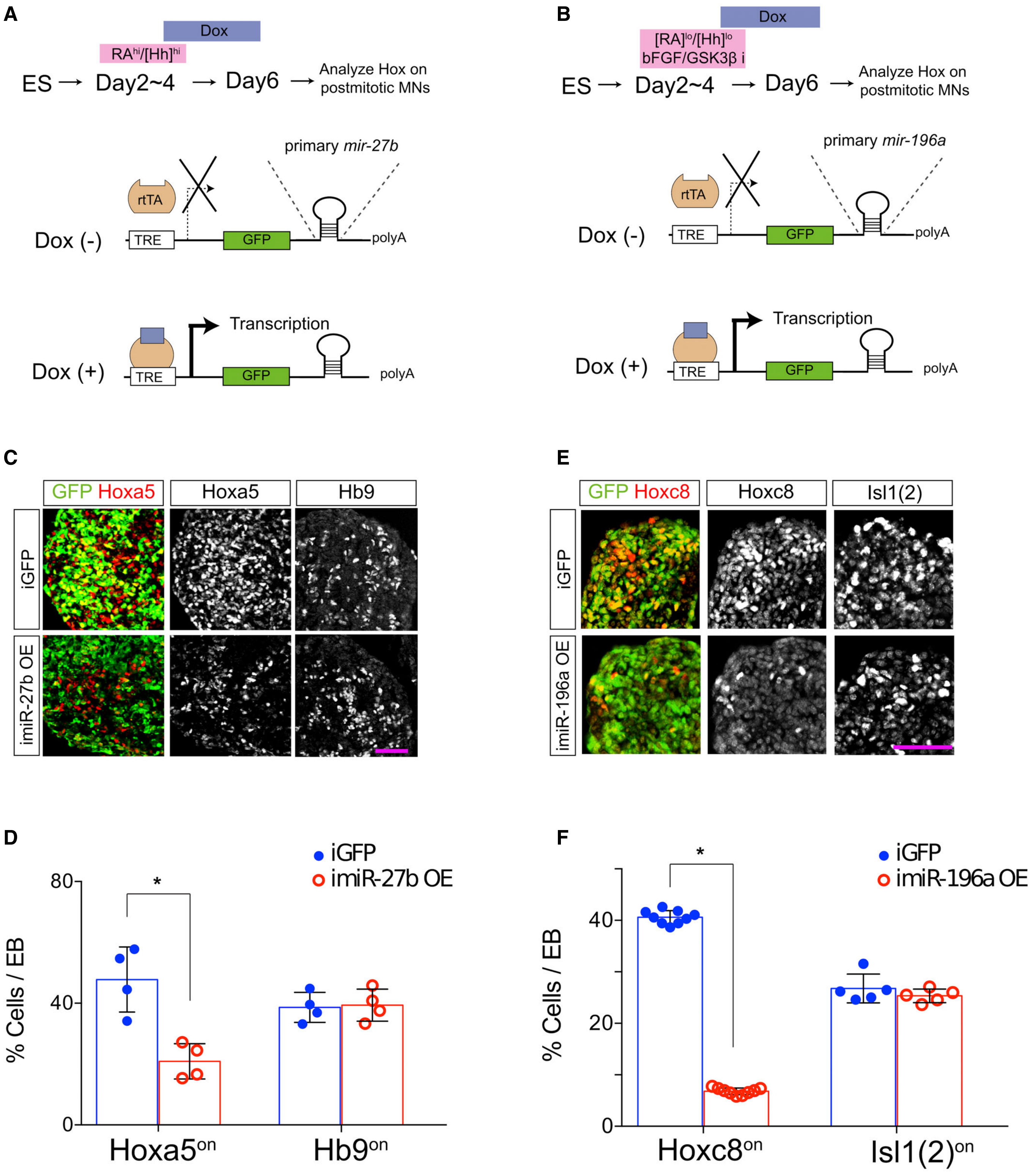

Figure EV5.

© 2021 The Authors
