# Supplementary material for: MicroRNA governs bistable cell differentiation and lineage segregation via a noncanonical feedback
Source: Mol Syst Biol. 2021 Apr 23;17(4):e9945. doi: 10.15252/msb.20209945 (PMC8062999; doi:10.15252/msb.20209945)
Supplement: Supplementary file 4 — Movie EV2 [file MSB-17-e9945-s002.zip › Movie EV2 Legend.docx]

**Legends of Expanded View Movies**

**Movie EV2**. **Time course simulation of the T-UR Model**. A grid of 10×40 cells was used to represent a segment of developing spinal cord where progenitor cells are influenced by competing FGF and RA concentrations (Figure EV2 B). Heatmaps reflect final distributions of denoted molecules in the tissue domain. See Appendix Supplementary Methods for details of the T-UR Model, and Figure EV2 E for the network diagram.
