# Supplementary material for: MicroRNA governs bistable cell differentiation and lineage segregation via a noncanonical feedback
Source: Mol Syst Biol. 2021 Apr 23;17(4):e9945. doi: 10.15252/msb.20209945 (PMC8062999; doi:10.15252/msb.20209945)
Supplement: Supplementary file 8 — Movie EV6 [file MSB-17-e9945-s006.zip › Movie EV6 Legend.docx]

**Legends of Expanded View Movies**

**Movie EV6**. **Time course simulation of the mmi-S Model under miR-27-KO condition**. A grid of 10×40 cells was used to represent a segment of developing spinal cord where progenitor cells are influenced by competing FGF and RA concentrations (Figure EV2 B). Heatmaps reflect final distributions of denoted molecules in the tissue domain. The production rate of miR-27 was set to zero in all cells. See Appendix Supplementary Methods for details of the mmi-S Model, and Figure 7A for the network diagram.
